# Shifts in coastal sediment oxygenation cause pronounced changes in microbial community composition and associated metabolism

**DOI:** 10.1186/s40168-017-0311-5

**Published:** 2017-08-09

**Authors:** Elias Broman, Johanna Sjöstedt, Jarone Pinhassi, Mark Dopson

**Affiliations:** 1 Centre for Ecology and Evolution in Microbial Model Systems (EEMiS), Linnaeus University, Kalmar, Sweden; 2 Present address: Department of Biology/Aquatic ecology, Lund University, Sölvesgatan 37, 223 62 Lund, Sweden; 3 Present address: Centre for Ocean Life, Institute for Aquatic Resources, Technical University of Denmark, 2900 Charlottenlund, Denmark

**Keywords:** 16S rRNA, Anoxic, Oxic, Metagenomics, Metatranscriptomics, Sediment

## Abstract

**Background:**

A key characteristic of eutrophication in coastal seas is the expansion of hypoxic bottom waters, often referred to as ‘dead zones’. One proposed remediation strategy for coastal dead zones in the Baltic Sea is to mix the water column using pump stations, circulating oxygenated water to the sea bottom. Although microbial metabolism in the sediment surface is recognized as key in regulating bulk chemical fluxes, it remains unknown how the microbial community and its metabolic processes are influenced by shifts in oxygen availability. Here, coastal Baltic Sea sediments sampled from oxic and anoxic sites, plus an intermediate area subjected to episodic oxygenation, were experimentally exposed to oxygen shifts. Chemical, 16S rRNA gene, metagenomic, and metatranscriptomic analyses were conducted to investigate changes in chemistry fluxes, microbial community structure, and metabolic functions in the sediment surface.

**Results:**

Compared to anoxic controls, oxygenation of anoxic sediment resulted in a proliferation of bacterial populations in the facultative anaerobic genus *Sulfurovum* that are capable of oxidizing toxic sulfide*.* Furthermore, the oxygenated sediment had higher amounts of RNA transcripts annotated as *sqr*, *fccB*, and *dsrA* involved in sulfide oxidation. In addition, the importance of cryptic sulfur cycling was highlighted by the oxidative genes listed above as well as *dsvA*, *ttrB*, *dmsA*, and *ddhAB* that encode reductive processes being identified in anoxic and intermediate sediments turned oxic. In particular, the intermediate site sediments responded differently upon oxygenation compared to the anoxic and oxic site sediments. This included a microbial community composition with more habitat generalists, lower amounts of RNA transcripts attributed to methane oxidation, and a reduced rate of organic matter degradation.

**Conclusions:**

These novel data emphasize that genetic expression analyses has the power to identify key molecular mechanisms that regulate microbial community responses upon oxygenation of dead zones. Moreover, these results highlight that microbial responses, and therefore ultimately remediation efforts, depend largely on the oxygenation history of sites. Furthermore, it was shown that re-oxygenation efforts to remediate dead zones could ultimately be facilitated by in situ microbial molecular mechanisms involved in removal of toxic H_2_S and the potent greenhouse gas methane.

**Electronic supplementary material:**

The online version of this article (doi:10.1186/s40168-017-0311-5) contains supplementary material, which is available to authorized users.

## Background

Dissolved oxygen in the oceans has declined in the past five decades, and the decrease is accelerated by an increase in temperature. In conjunction with this decline, an expansion of oxygen-minimum-zones in the oceans has been observed. With an increase in ocean water temperature, climate change is suggested to further enhance this decrease of oxygen [1]. The expansion of episodic and persistent hypoxic areas, often referred to as ‘dead zones’, is altering ecosystem functioning of coastal systems largely as a result of eutrophication [2]. The negative influence of hypoxia on higher trophic levels is evident by damage to fisheries via increased fish mortality [3]. The provision of nutrients from eutrophication events fuels phytoplankton blooms that eventually die, and a portion of the biomass sinks to the sediment surface where it is degraded by aerobic microbes [4]. These metabolic processes deplete the available oxygen [5] such that the sediments and the overlying bottom water become anoxic. The oxygen depletion of sediments inhibits life for many macro and microorganisms, although anaerobic microbes still thrive (e.g., [6–8]). Given the importance of oxygenation levels for chemical fluxes between the sediment-water interface and the preference of many microorganisms for either oxic or anoxic conditions [9], it is expected that many (bio)chemical pathways mediated by microorganisms are significantly affected by oxygen fluctuations. However, details on the molecular mechanisms regulating biogeochemical energy and nutrient cycles under fluctuating oxygen conditions remain largely unexplored.

In the Baltic Sea, hypoxic (<2 mg/L) sediment zones that persist during the whole year are currently about 10-fold more widespread compared to 100 years ago [10]. The area of Baltic Sea episodic hypoxic and anoxic coastal zones has also increased during the last decades. Oxygen availability in Baltic Sea dead zones fluctuates over time due to the inflow of high saline, oxygen-rich water. The occurrences of these inflows are less common compared to three decades ago, and many previously oxygenated bottom sediments are instead rich in hydrogen sulfide (H_2_S) [11].

Microbial communities in sediments are stratified due to the availability of electron acceptors that are utilized according to the energy gain, from most to least [6, 12]: O_2_, NO_3_^−^, Mn^3+^ and Mn^4+^ oxides, Fe^3+^ oxides, and SO_4_^ 2−^. This use of alternative electron acceptors in turn influences the oxygen-reduction potential at various depths in the sediment. Analysis of sediments from around the world has identified key microbial taxa associated with these redox reactions [13–15]. The microbial reduction of Fe^3+^ and SO_4_^ 2−^ produces ferrous iron (Fe^2+^) and toxic H_2_S, respectively, that can subsequently chemically precipitate as iron sulfides. This gives anoxic sediments their distinctive black color [6]. Conversion of previously oxic sediments to anoxic conditions would result in the production of H_2_S while re-oxygenation of these areas would potentially detoxify the H_2_S and facilitate the re-establishment of benthic micro and macroorganisms. Knowledge of how microbial community composition changes during fluctuations in oxygen levels would aid in interpreting the consequences of bulk chemical changes in the sediment.

The dominant microbial processes in anoxic sediments are heterotrophic/autotrophic sulfate reduction with methanogenesis occurring below the SO_4_^ 2−^ reduction zone [6]. The reduced products (Mn^2+^, Fe^2+^, and S^2−^) are released into the sediment pore-water where they diffuse upwards and can be consumed, e.g., by aerobic H_2_S and Fe^2+^ oxidation or anaerobic denitrification [6, 16, 17]. These processes form vertical redox zones in the sediment that may constitute just a few hundred μm [5, 18, 19]. Lower dissolved oxygen concentrations in the overlying water also cause redox layers to migrate upwards in the sediment [20]. Due to the difference in energy yield from available oxidations, organic matter is preserved in oxygen deficient sediments [21], and its degradation is slower compared to oxic sediments [22, 23]. Although it is known that the old and buried organic matter is rapidly degraded upon re-oxygenation, the time scales required to remove the organic matter and to detoxify the waters remain little studied.

Microbial communities in anoxic marine sediments are rich in *Gamma* and *Deltaproteobacteria* related to sulfate reduction (e.g., [24, 25]). In addition, identification of the microbial community structure in O_2_ rich, hypoxic, and anoxic surficial sediments has been conducted worldwide, including in the Baltic Sea (e.g., [7, 26–28]). Identification of sediment microbial communities along an eutrophication gradient in the Baltic Sea reveals patterns among major microbial groups and that *Planctomycetes* and *Betaproteobacteria* were more abundant in oxygen rich compared to oxygen deficient sediments [27]. In general, how the majority of the microbial community and its metabolism adapts to in situ shifts in oxygen concentration has not been identified.

In this study, we used complete sediment cores in a novel oxygen shift experimental design to investigate the impact of altering oxidative and reductive conditions in coastal Baltic Sea sediments exposed to seasonal transitions, i.e., oxic all-year-round, anoxic benthic water all-year-round, and an area that alternates between oxic and anoxic depending on the time-of-year. The aims of the study were (1) to link the microbial community structure to changing metabolic pathways in the sediment surface and benthic water as a result of oxygen shifts; (2) to investigate if associated shifts in metabolic strategies and functions regulate chemical fluxes; and (3) to elucidate how the chemical fluxes are affected by the oxygen in the benthic water and sediment surface.

## Methods

### Sampling and handling of sediment cores

Sampling was conducted in a Baltic Sea bay close to Loftahammar, Sweden on November 18, 2013. The studied bay was chosen as it contained sampling sites with different oxygenation histories based upon previous measurements of dissolved bottom water oxygen in the area and an earlier pilot study (full details in Additional file 1: Text S1). At the time of sampling, three sites were chosen: (1) a 6.5-m-deep (from water to sediment surface) site with long-term oxic sediment (brown sediment), 6.5 °C and 11.3 mg/L O_2_ in the bottom water, located at WGS 57 53.214, 16 35.934; (2) a 31-m-deep site with long-term anoxic sediment (black sediment with a strong smell of H_2_S), 2.6 °C and 0.85 mg/L O_2_, WGS 57 53.531, 16 35.165; and (3) a 21-m-deep site in an intermediate zone between the two (predominantly black sediment with patches of brown), 2.8 °C and 0.8 mg/L O_2_, WGS 57 53.545, 16 35.476.

Sediments were sampled using a gravity corer (60-cm-long polymethylmethacrylate tubes with an inner diameter of 7 cm). The sediment (27.5 to 33.5 cm height) was collected with the overlying bottom water. Sediment cores were sealed at the top and bottom with polyoxymethylene lids with nitrile o-rings. To determine the chemistry and the microbial communities in the sediment at the time of sampling, three sediment cores were immediately tested for each site (nine in total). Bottom water overlying the sediment (approximately 50 cm above) was collected using a 2 L Ruttner water sampler (SWEDAQ HB). Bottom water temperature and dissolved oxygen were measured in the field using in situ sensors (WTW Multiline; full details in Additional file 1: Text S1). From the overlying water, 50 mL was aseptically transferred into a sterile tube and kept on ice for subsequent DNA extraction. In addition, a 15 mL sterile tube was filled to the cap and stored on ice for measurement of Fe^2+^, Fe^3+^, SO_4_^2−^, PO_4_^3−^, NO_2_^−^ in conjunction with NO_3_^−^, reduced sulfur compounds, pH, and redox potential. The cores were transported to the laboratory at ~7 °C and were stored closed for 1 day in incubation temperature (8 °C) prior to start of the experiments. An overview of the chemistry and nucleic acid extractions conducted during the incubation is presented below. To verify the microbial community composition responses to oxygenation, complementary samples for 16S rRNA gene operational taxonomic unit (OTU) analysis were collected during an experiment in May 2014 by sampling sediment cores from the anoxic field site, out of which five cores were maintained anoxic and five cores were turned oxic (full details of sampling and incubation procedure in [29]).

### Incubation of sediment cores

Sixteen sediment cores were incubated in darkness for 21 days at 8 °C. The cores were incubated at 8 °C as this was similar to the surface water temperature of 6.5 °C and re-oxygenation of the anoxic sediments occurs naturally by complete mixing of the water column or artificially by pumping of surface waters to the sea floor. Cores turned or maintained oxic were oxygenated by aerating the overlying water on top of the sediment during the whole experiment. This resulted in ~10 mg/L O_2_ in the water phase of the ‘anoxic-to-oxic’ (*n* = 3), intermediate-to-oxic (*n* = 3), and oxic control (*n* = 2). Cores were turned or maintained anoxic by bubbling the overlying water with N_2_-gas for 45 min at the start of the incubation. This resulted in O_2_ concentrations below 0.1 mg/L (oxygen electrode, Innovative Instruments in the ‘oxic-to-anoxic’ (*n* = 2), intermediate-to-anoxic (*n* = 3), and ‘anoxic control’ (*n* = 2). To ensure that water sub-sampled for chemistry measurements and DNA extraction was homogenized, the water phase in each sediment core was mixed using magnets in sterile tubes suspended in the water that were agitated using magnets rotating outside the sediment cores. The water phase was sub-sampled approximately every fourth day throughout the incubation, while the 0–1 cm sediment surface was sliced after 21 days of incubation.

### Sub-sampling and chemistry measurements

Sub-sampling of the anoxic cores briefly exposed the water surface to oxygen and therefore, they were subsequently bubbled with N_2_-gas for 1 to 5 min and the O_2_ concentration in the water phase confirmed to be <0.1 mg/L. That the mixing and aeration methodology oxygenated the water phase and surficial sediment was confirmed in eight separate oxic control sediment cores with an identical set-up using an optical oxygen meter attached to a motorized micromanipulator (FireStingO2; OXR50 oxygen sensor; Micromanipulator MU1, Pyroscience), and further verified in another experiment conducted during May 2014 using a similar incubation setup (see [29]). The oxygen penetration depth in the sediment surface was not measured in this experiment, but was confirmed to be ~1.5 mm in an additional experiment conducted in April 2016 (15 days of incubation with a similar setup at 8 °C in darkness; determined with the optical oxygen meter attached to the motorized micromanipulator).

Sub-samples taken for analysis were (1) 4.5 mL sediment mixed with 0.5 mL 5% water-saturated phenol in absolute ethanol [30], flash frozen in liquid nitrogen, and later used to extract RNA; (2) 10 mL sediment was stored on ice and used for DNA extraction (16S rRNA gene and metagenomics); (3) 5 mL sediment used for total pore-water iron; (4) 1 mL sediment transferred into a pre-weighed 2 mL polypropylene tube used to estimate sediment organic matter (% wt); and (5) the remaining sediment was retained for measurement of SO_4_^ 2−^, PO_4_^ 3−^, NO_2_^−^ + NO_3_^−^, reduced sulfur compounds, pH, and redox potential. Sediment slices for DNA and RNA were stored at −80 °C until extracted. 50 mL of water for DNA extraction was filtered through a 0.2 μm pore size Supor-200 25 mm filter (PALL Corporation), the filter transferred to a sterile tube, and stored at −80 °C until extraction. A schematic overview of the nucleic acid extractions and chemistry measurements are given in Additional file 1: Text S1.

Water samples were filtered through a 0.7 μm pore size glass fiber 25 mm filter (GF/F filter, Whatman). PO_4_^ 3−^, NO_2_^−^, + NO_3_^−^ were measured with a DR 5000 Hach-Lange spectrophotometer according to Valderrama JC [31]. SO_4_^ 2−^ was also measured on the Hach-Lange spectrophotometer utilizing the LCK 353 kit (Hach-Lange). Fe^2+^ and Fe^3+^ were measured with a SmartSpec 3000 Bio-Rad spectrophotometer according to Dawson MV and Lyle SJ [32]. pH (pHenomenal, VWR pH electrode) and redox potential (Ag^0^/AgCl SI Analytics electrode, Mettler Toledo) were also measured. Frozen water and sediment pore-water samples were subsequently used to measure reduced sulfur compounds (tetrathionate and thiosulfate) by cyanolysis [33] according to Kelly DP, Chambers LA, and Trudinger PA [34]. Sediment organic matter (% wt) was calculated by heating crucibles in a furnace at 550 °C for 30 min (OWF 1200, Carbolite), the crucibles were weighed, dry sediment samples added (dried at 80 °C for 3 days), weighed again, heated at 550 °C for 4 h, and finally weighed to determine the percentage organic matter. The rate of organic matter degradation was estimated by dividing the sediment percentage organic matter by the amount of incubated days. Sediment pore-water samples were centrifuged at 2200 g for 15 min, the supernatant filtered through a 0.7 μm pore size glass fiber GF/F 25 mm filter, and homogenized before being analyzed as described above.

### DNA and RNA extraction, sequencing, and bioinformatic analysis

DNA was extracted from frozen water phase filters according to Boström KH, Simu K, Hagström Å, and Riemann L [35] and stored at −20 °C until Illumina library preparation. DNA and RNA extractions from homogenized sediment samples were carried out using the PowerMax Soil DNA and PowerSoil RNA kits (MO BIO Laboratories), respectively. RNA samples were DNase treated twice using the Turbo DNA-free kit (Ambion). After DNase treatment, rRNA was removed using the Ribominus Transcriptome Isolation Kit (Yeast and Bacteria version; Invitrogen Life Technologies). DNA and RNA concentrations were measured using a NanoDrop 2000 and a Qubit 2.0.

The 16S rRNA Illumina library was prepared using PCR primers 341f and 805r [36] and a modified PCR program by Hugerth LW, Wefer HA, Lundin S, Jakobsson HE, Lindberg M, Rodin S, Engstrand L, and Andersson AF [37]. PCR program modification and the processes of adding Illumina adapters and indexes were conducted according to Lindh MV, Figueroa D, Sjöstedt J, Baltar F, Lundin D, Andersson A, Legrand C, and Pinhassi J [38]. Samples were sequenced at Science for Life Laboratory (SciLifeLab), Stockholm using the Illumina MiSeq platform. Most water samples were sequenced with 2 × 201 bp pair-ends while sediment samples (and the remaining water samples at time point day 12) were sequenced with 2 × 301 bp pair-ends. The 16S rRNA gene sequence data were analyzed and sequences clustered into OTUs (97% similarity) according to UPARSE pipeline [39], as described on the BILS website for using UPARSE on the UPPMAX cluster (available with all scripts at: https://wiki.bils.se/wiki/Running_the_Uparse_pipeline_at_the_UPPMAX_cluster). After merging of pair-ends and quality filtering, the sediment samples had an average sequence count of 56,271 reads (min 26,712; max 376,267) while the water samples had an average sequence count of 40,335 reads (min 1118; max 83,223). Two water samples, oxic field and one anoxic-to-oxic replicate at the end of the experiment were removed due to a final sequence count below 1000. OTUs were taxonomically annotated with a 95% sequence identity threshold against the SSU Ref NR 99 v119 SILVA database [40]. The final sequence data was analyzed using Explicet [41]. Rarefraction curves of reads and OTUs were created with the vegan package in R (reads were subsampled to the lowest sample size). Phylogenetic analysis was conducted by ClustalW sequence alignment and construction of maximum-likelihood trees using MEGA 6 and 7 [42, 43]. Habitat specialization was calculated for water samples and their respective sediment samples (field and sliced at the end of the experiment after 21 days) according to Pandit SN, Kolasa J, and Cottenie K [44] using Levin’s niche width (B) index:

1 $$ B=1/{\sum}_{i=1}^N{p}_{ij}^2 $$

where pij is the proportion of OTU *j* in the sample *i* and *N* is the number of samples. OTUs with high *B* are classified as habitat generalists and are evenly distributed along a wide range of habitats. In contrast, OTUs with a low B are considered habitat specialists and are unevenly distributed among sampling sites. An average niche width was calculated for each sample, to investigate differences in average niche between treatments. A full list of reads obtained from the sequencing facility after merging and quality trimming as well as the clustered OTUs is given in Additional file 2: Table S1.

Extracted community DNA from the end of the oxic-to-anoxic (*n =* 2), intermediate-to-oxic (*n =* 2), and anoxic-to-oxic sediment (*n =* 2) experiments were sequenced at SciLifeLab, Stockholm using Illumina MiSeq pair-ends 2 × 301 bp (average 7.8 million read pairs per sample). Metagenomic samples were analyzed according the SciLife’s Metagenomic Assembly Workshop 2014 (available with all scripts at: http://2014-5-metagenomics-workshop.readthedocs.io/en/latest/assembly/). In detail, Illumina universal adapter and low quality sequences after the adapter were cut from reads using default settings of cutadapt 1.8.0 [45] and then were quality trimmed using sickle 1.210 with default settings. After adapter removal and trimming, approximately 98% of reads remained with an average length of 155 bp. 16S and 18S sequences were extracted with SortMeRNA v2.1 [46], and the SILVA SU Ref v 128 database [40] while sequences below 100 bp were removed using fastx_clipper in the FASTX-toolkit. The 16S and 18S sequences were then clustered for OTUs, reads mapped, and results analyzed in the same manner as the 16S rRNA gene amplicon data. Assembly of the metagenomic data was carried out with a kmer size of 31, 41, 51, 61, 71, and 81 using Ray 2.3.1 [47]. Due to insufficient sequence coverage, only a kmer size of 31 was used and after assembly there was on average 3.4 million contigs with an average length of 193 bp and maximum 14,744 bp. The assembly was annotated using the PROKKA pipeline [48], in which genes were annotated against the UniprotKB/Swiss-Prot database for functional genes and reference taxonomical organisms. Reads were mapped onto the assemblies using samtools 1.1 [49] and Bowtie2 2.2.3 [50] in conjunction with Picard tools 1.77 (Broad Institute). On average, 35% of the reads were mapped back onto the assemblies and the counts per gene per was then calculated using BEDTools 2.23 [51]. The final counts were normalized and expressed as counts per million reads (CPM; i.e., relative proportion × 1,000,000). A full list of reads obtained from the sequencing facility after adapter and quality trimming, the amount of contigs, and their length with N50 values is available in Additional file 2: Table S1.

In total, 11 DNase treated and rRNA depleted RNA sediment samples from the end of the experiment were sequenced: oxic-to-anoxic (*n =* 2), intermediate-to-oxic (*n =* 2), intermediate-to-anoxic (*n =* 2), anoxic-to-oxic (*n =* 2), oxic control (*n =* 1), and anoxic control (*n =* 2). The samples were sequenced at SciLifeLab, Stockholm using Illumina HiSeq pair-ends 2 × 126 bp. Illumina universal adapters and low quality sequences following the adapter sequence were cut from reads using SeqPrep with default settings. Sequences were then quality trimmed using Trimmomatic 0.32 with settings LEADING:5 TRAILING:5 MINLEN:36 [52]. This resulted in an average of 19 million read pairs per sample. Due to insufficient sequence coverage of the metagenomic samples, a de novo assembly was chosen for the RNA data. De novo assembly was conducted using Trinity with default settings [53] producing 185,092 contigs of average length 389 bp and maximum length 17,997 bp. After assembly, the Trinotate pipeline was followed (available with all scripts at: https://trinotate.github.io/). The assembly was annotated with BLASTX and BLASTP using the UniProtKB/Swiss-Prot and PFAM databases followed by mapping RNA transcripts and differential expression analysis. rRNA sequences were identified with RNAmmer 1.2. Reads were mapped back onto the assembly using RSEM [54] in conjunction with samtools 1.1 [49] and Bowtie2 2.2.3 [50]. Differential expression was calculated using the package edgeR [55] in the R software suit Bioconductor 3.2. The annotation and differential expression data was loaded into a SQLite Trinotate 2.0.2 database file and was exported as matrix tables. The final differential expression matrix containing all samples was used to cluster significantly expressed RNA transcripts using Euclidean distance clustering with a *p* < 0.001 and minimum fold change of four. Annotation of significantly expressed transcripts against the PFAM database was manually compared to that of UniProtKB/Swiss-Prot and showed overall similar results. As more RNA transcripts could be annotated with UniProtKB/Swiss-Prot only annotations from that database are reported. Count values are expressed as trimmed mean of *M* values (TMM) normalized Fragments Per Kilobase Million (FPKM) units. The reference organisms in the UniprotKB database linked to annotated genes were used to infer the active microbial community composition (expressed as proportion (%) of FPKM). For significantly expressed RNA transcripts, from edgeR analysis, annotated against UniProtKB/Swiss-Prot, the Uniprot IDs with similar names were merged. The Uniprot IDs that could then be linked to a KEGG Orthology (KO) identifier were merged based on KO name (709 UniProt IDs could be linked). The remaining Uniprot IDs with at least 100 FPKM for any sample were merged by gene name (yielding a total of 157 statistically significant genes). A full list of reads obtained from the sequencing facility and the amount of contigs and their length with N50 values is available in Additional file 2: Table S1. To link the significant genes to a taxonomic affiliation, RNA transcript sequences were annotated using BLAST with five hits against the UniProtKB/Swiss-Prot database. The top hit reference organism available in the UniProtKB/Swiss-Prot database was used in conjunction with the FPKM values for each sample to calculate proportion of taxonomic affiliation, while the remaining hits were used to interpret the data in greater detail.

All samples were sequenced at Science for Life Laboratory, Stockholm using either the Illumina MiSeq or HiSeq platforms. A full list of reads for the various sequencing programs is given in Additional file 2: Table S1. The nucleic acid sequences have the NCBI BioProject accession number: PRJNA322450.

## Results

### Chemical fluxes in the water phase and sediments

**Fig. 1:**
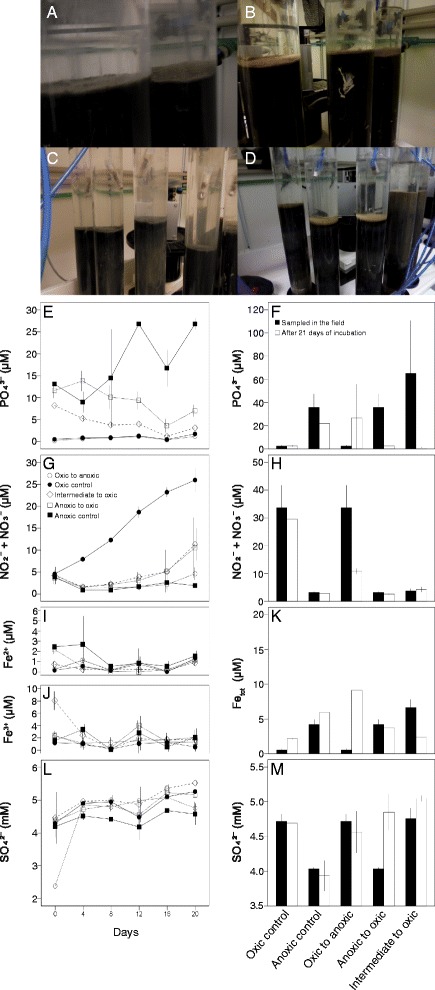
Chemistry data from the incubation experiments. The pictures show **a** anoxic cores on the first day of incubation; **b** anoxic cores turned oxic after 21 days of incubation; **c** intermediate cores on the first day of incubation (first three from left side) and anoxic cores on the first day of incubation; and **d** intermediate cores turned oxic after 21 days of incubation (first three from left side) and oxic control cores maintained oxic after 21 days of incubation. Measurements of chemistry from the water phase are shown on the left **e** PO_4_^ 3−^, **g** NO_2_^−^ + NO_3_^−^, **i** Fe^2+^, **j** Fe^3+^, and **l** SO_4_^2^^−^ (symbols: *black circles*, oxic control; *black squares*, anoxic control; *white circles*, oxic-to-anoxic; *white squares*, anoxic-to-oxic; and *white diamonds*, intermediate-to-oxic); pore-water chemistry from the top 1-cm-sliced sediment are on the right **f** PO_4_^ 3−^, **h** NO_2_^−^ + NO_3_^−^, **k** total iron (Fe_tot_), and **m** SO_4_^ 2−^ (*black bars* denote zero time point measurements from the field and *white bars* denote measurements at the end of the incubation experiment). All values are averages of triplicates ±1 SD, except water and sediment anoxic-to-oxic (*n =* 2) and sediment anoxic-to-oxic (*n =* 2)

Upon oxygenation of anoxic sediments, a ~0.5-cm light brown surface layer developed (Fig. 1b, d), which indicated that reduced iron had oxidized and iron sulfides no longer colored the sediment black. The anoxic control water phase phosphate (PO_4_^3−^) concentration increased from 13.1 ± 0.2 μM on day 0 to 26.8 ± 1.3 μM on day 20 (number of replicates (*n*) = 2, SD = 1) compared to a decrease over the incubation time in all the other cores (Fig. 1e and details in Additional file 3: Table S2). This decrease was likely due to precipitation of PO_4_^3−^ coupled to Fe^3+^ and/or PO_4_^3−^ uptake by microorganisms. The PO_4_^ 3−^ concentrations in the surface sediment (top 1 cm) pore-water had high variability among cores although the general trend was of a decreasing concentration over time in the anoxic-to-oxic and intermediate-to-oxic incubations (Fig. 1f). This variation could potentially be due to H_2_S interference with the analysis [31] that was also observed in a similar experiment [29]. In contrast, the PO_4_^ 3−^ concentration in the oxic sediment sliced and sampled in the field (2.4 ± 0.8 μM, *n =* 3) rose to 26.6 ± 29.1 μM in the oxic-to-anoxic incubation after 20 days (*n =* 2; Fig. 1f). In the anoxic-to-oxic and intermediate-to-oxic cores these trends were potentially due to PO_4_^ 3−^ uptake by microorganisms and oxidation of Fe^2+^ to Fe^3+^ resulting in Fe-bound non-soluble PO_4_^3−^ and a ~0.5-cm light brown layer on the sediment surface (Fig. 1b). In the oxic-to-anoxic and intermediate-to-anoxic cores these trends were likely due to reduction of Fe^3+^ causing coupled PO_4_^ 3−^ to become soluble in the pore-water. The opposite trend occurred for the surface sediment NO_2_^−^ + NO_3_^−^ as the concentration decreased over time in the oxic-to-anoxic cores (Fig. 1h). Also in the water phase, the NO_2_^−^ + NO_3_^−^ concentration decreased over time except in the oxic control where it increased from 4.6 ± 1.0 on day 0 to 26.0 ± 7.8 μM on day 20 (*n =* 2; Fig. 1g).

The sediment pore-water sulfate concentration was higher in the oxic control compared to the anoxic control (Fig. 1m), presumably as sulfate reduction in the anoxic sediment converted sulfate to sulfide. This trend was replicated in the anoxic-to-oxic sediment where the sulfate concentration increased as anaerobic sulfate reduction was likely replaced by reduction of oxygen (Fig. 1m). Inorganic sulfur in the water phase (tetrathionate and thiosulfate) remained low throughout the experiment with no statistically valid differences among the treatments (*p* > 0.05; one-way ANOVA). The thiosulfate concentration was zero in all samples except in the sediment pore-water from the anoxic cores sliced in the field that had 48 ± 15 μm (*n =* 2; Additional file 3: Table S2).

The organic matter content in the oxic field sample was 11.7 ± 1.0% (*n =* 3) which was lower than the anoxic (18.4 ± 0.5%, *n =* 3) and intermediate (18.5 ± 0.6%, *n =* 2) field sediments (Table 1). The oxic and intermediate organic matter content remained stable over the experiment while the anoxic-to-oxic organic matter significantly decreased to 15 ± 1.4% (one-way ANOVA, F = 28.41, *p* < 0.01, *n =* 3). There was also a statistically significant decrease when the anoxic-to-oxic organic matter decrease was compared to the anoxic control that had 19.0 ± 0.3% (F = 27.48, *p* < 0.05, *n =* 2).

**Table 1:** Organic matter (%) determined by loss on ignition, on sediment sampled in the field and after 21 days of incubation in darkness after either turned/maintained anoxic/oxic. All values are averages of triplicates (SD = 1) except intermediate field, oxic control, and anoxic control (all *n* = 2)

Sampling site	Field	Turned/kept anoxic	Turned/kept oxic
Long-term oxic	11.7 ± 1.0	12.5 ± 0.4	13.1 ± 0.1
Intermediate zone	18.5 ± 0.6	18.1 ± 1.4	18.7 ± 0.4
Long-term anoxic	18.4 ± 0.5	19.0 ± 0.3	15.0 ± 1.4

### Identification of microbial communities

**Fig. 2:**
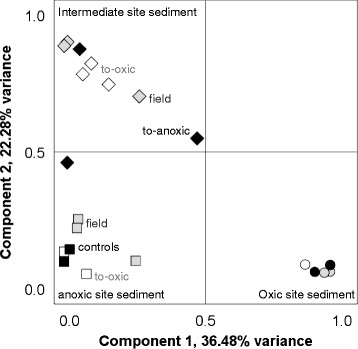
Principal component analysis of the OTU communities in the top 1-cm sediment layer. Symbols: *gray circles*, oxic field; *black circles*, oxic control; *white circles*, oxic-to-anoxic; *gray squares*, anoxic field; *black squarse*, anoxic control; *white squares*, anoxic-to-oxic; *gray diamonds*, intermediate field; *white diamonds*, intermediate-to-oxic; and *black diamonds*, intermediate-to-anoxic

Rarefraction curves of sequence counts for the 16S rRNA gene OTUs showed that most of the microbial diversity had been covered (Additional file 4: Figure S1). A principal component (PCA) analysis based on the relative abundance 16S rRNA gene OTUs showed that the oxic sediment samples from both the field and at the end of the experimental incubations were tightly clustered (Fig. 2). In contrast, the samples from the anoxic and intermediate sediment cores were more dispersed after the re-oxygenation treatment. Alpha diversity calculations on all OTUs using Shannon’s H index showed a significantly higher diversity in the oxic samples (9.56 ± 0.11, *n* = 7), compared to intermediate (8.97 ± 0.28, *n* = 8) and anoxic (8.75 ± 0.32, *n* = 7) samples (one-way ANOVA; oxic and intermediate *F* = 22.63, *p* < 0.01, and oxic and anoxic samples *F* = 26.72, *p* < 0.01; also significantly different with non-parametric Kruskal-Wallis tests (*p* < 0.01)). Levin’s niche width (*B*) index was used to analyze the OTU uniformity of distribution between sites, with high values indicating habitat generalists while a low *B* indicates habitat specialists. All values suggested a majority of generalists although the intermediate sediment samples showed statistically significant higher values (*B* = 8.11 ± 0.27, *n =* 9) compared to the oxic samples (7.67 ± 0.28, one-way ANOVA, *F* = 7.85, *p* < 0.05, *n =* 7). The anoxic sediment samples had a *B* value of 7.88 ± 0.31 (*n =* 7), and the differences were non-significant with the other groups.

**Fig. 3:**
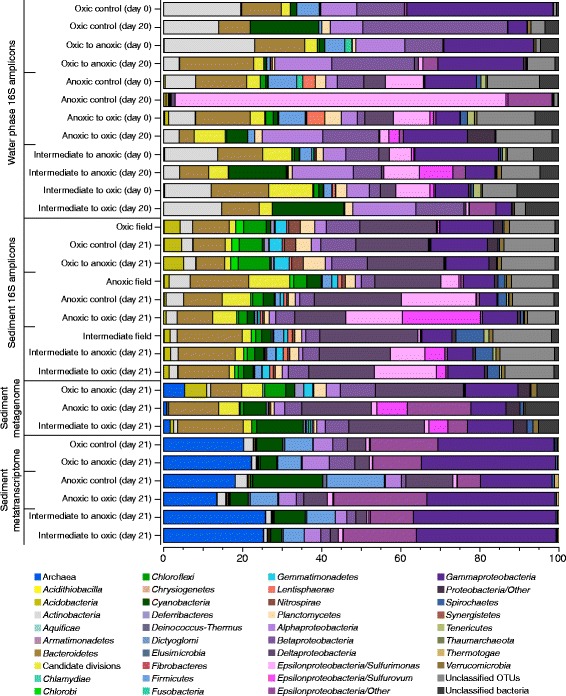
Stacked bar graphs of microbial community composition from the start and the end of the incubation experiment. The partial 16S rRNA gene amplicons show relative abundance (%) of counts. Taxonomic affiliation was linked to the metagenome by extracting 16S rRNA gene sequences from the metagenome dataset (relative abundance (%) of counts is shown). The community composition identified based on the metatranscriptome was derived from the taxonomy affiliated with annotated genes in the metatranscriptomic dataset (proportion (%) of FPKM). The *Proteobacteria* are divided into classes and *Epsilonproteobacteria* has been further divided into *Sulfurimonas*, *Sulfurovum*, and other *Epsilonproteobacteria*

The dominant taxa in the microbial communities from the oxic, intermediate, and anoxic sediments were affiliated with the *Delta-*, *Epsilon-*, and *Gammaproteobacteria* plus *Bacteroidetes* (Fig. 3). 5952 OTUs (constituting 95.9% of the total) in the sediment phases at the end of the experiment were relatively stable compared to the field sediments (i.e., changed <0.1% in relative abundance). In contrast, more than a 0.1% increase or decrease in relative abundance of OTUs between the field sediments and the end of the incubations was recorded for 246 OTUs. These changing OTUs represented 47–61% reads in the initially anoxic sediment, 52–57% in the intermediate, and 22–25% in the initially oxic sediment. The changing OTUs primarily belonged to the *Sulfurimonas* and *Sulfurovum* genera (*Epsilonproteobacteria*)*,* but also included, e.g., *Desulfobacula*, *Spirochaeta*, and unclassified OTUs (Additional file 5: Figure S2). Consistent with the tightly clustered oxic samples in the PCA (Fig. 2), the relative abundance of the dominant OTUs in the cores from the oxic field samples and after incubations had ≤2-fold difference between all the treatments. These dominant populations included OTUs affiliated with the genera *Desulfobulbus*, *Nitrosomonadaceae*, *Anaerolineaceae*, and *Gemmatimonadaceae* (Additional file 5: Figure S2 and Additional file 6: Data S1). In the sediment cores from the intermediate site, there was a stronger microbial community shift compared to the oxic cores, with *Sulfurimonas*-like OTUs increasing from <1% in the field samples to a relative abundance of 14.4 and 8.4% in the intermediate-to-oxic and intermediate-to-anoxic cores, respectively (Fig. 3 and Additional file 6: Data S1). Dominant individual *Sulfurimonas*-like OTUs in the intermediate-to-oxic cores increased up to 374-fold (Additional file 6: Data S1). In addition, *Sulfurovum*-like OTUs increased from <1% in the field to a relative abundance of 2.2 and 4.8% in the intermediate-to-oxic and intermediate-to-anoxic cores, respectively (Fig. 3), with one dominant *Sulfurovum*-like OTU in the intermediate-to-anoxic increasing 365-fold (Additional file 6: Data S1). In the sediment cores from the anoxic site, the relative abundance of *Sulfurimonas*-like OTUs increased from 4.5% in the anoxic field sample to 18.4% in the anoxic control and 13.8% in the anoxic-to-oxic cores (Fig. 3). Dominant single *Sulfurimonas*-like OTUs increased up to 414-fold in the anoxic control and up to 565-fold in the anoxic-to-oxic sediment (Additional file 6: Data S1). In contrast, the *Sulfurovum*-like OTUs decreased from 0.4% in the field samples to 0.2% in the anoxic control while increasing strongly to 19.0% in the anoxic-to-oxic treatment (Fig. 3 and Additional file 6: Data S1). The additional experiment conducted during May 2014 showed similar results regarding *Sulfurimonas*- and *Sulfurovum*-like OTUs when exposed to oxygen or maintained anoxic (Additional file 7: Figure S3).

Extracted 16S rRNA gene sequences from the metagenomic dataset at the end of the incubation showed a higher relative abundance of archaea in the sediment surface (up to 5% compared to 0.2% in the 16S amplicon data), possibly due to the primers used for PCR selecting against archaea [37]. The annotated metatranscriptomic dataset showed that archaea were active in the sediment at the end of the incubation and were represented up to 26% of the annotated data (proportion (%) of FPKM; Fig. 3). *Sulfurimonas*-like and *Sulfurovum*-like OTUs were present in the metagenomic dataset with the highest abundance in the anoxic-to-oxic sediment with 7.6 and 16.1%, respectively. However, these two *Epsilonproteobacteria* genera did not represent more than 1.4% in the metatranscriptome data. Instead, *Gammaproteobacteria* in the sediment had a proportion up to 36% in the metatranscriptome data at the end of the experiment (Fig. 3).

To investigate changes in microbial community composition due to chemical fluxes from the sediment surface, we also analyzed OTU data in the water phase of the incubated sediment cores. Microbial communities dominant in the water phase consisted of *Actinobacteria* (Fig. 3) (which is typical for the Baltic Sea [56]), with abundant OTUs primarily belonging to the orders *Acidimicrobiales*, *Corynebacteriales*, and *Frankiales* (Additional file 6: Data S1). Other dominant groups in the water phase were *Alpha*-, *Beta*-, *Epsilon*-, *Gammaproteobacteria*, and *Bacteroidetes*, along with unclassified OTUs (Fig. 3). In comparison to the sediment community, the water phase contained significantly higher relative abundance of *Alphaproteobacteria* (one-way ANOVA, *F* = 27.9, *p* < 0.01) and lower numbers of *Deltaproteobacteria* (one-way ANOVA, *F* = 465.9, *p* < 0.01). In the water phase there was a decreased relative abundance of OTUs affiliated with the *Actinobacteria* in the oxic-to-anoxic (22.5 to 3.9%) and intermediate-to-anoxic (13.0 to 4.0%; Fig. 3 and Additional file 6: Data S1). In contrast, an increase was observed in the *Cyanobacteria* (1.3 to 14.1%), *Alphaproteobacteria* (5.5 to 15.1%), and *Sulfurovum*-like OTUs (0.6 to 8.2%) (Fig. 3 and Additional file 6: Data S1). The relative abundance of *Sulfurimonas*-like OTUs strongly increased from 9.4 to 84.2% in the anoxic control water phase (Fig. 3). A decrease in *Bacteroidetes* OTUs (13.7 to 3.7%), order *Clostridiale*s (class *Firmicute*s, 4.9 to 0.5%), and *Lentisphaerae* (4.5 to 0.1%) occurred in the anoxic-to-oxic water phase (Fig. 3 and Additional file 6: Data S1).

### Correlations between microbial populations (OTUs) and chemical data

At the level of major taxa, the *Actinobacteria*, *Alpha*-, and *Betaproteobacteria* in the water phase were negatively correlated with PO_4_^ 3−^ (*p* < 0.01; *r* = −0.42, −0.37, and −0.44, respectively; Additional file 8: Table S3). *Cyanobacteria* and *Betaproteobacteria* in the water phase positively correlated with NO_2_^−^ + NO_3_^−^ while *Cyanobacteria* also had a positive correlation to SO_4_^ 2−^ in the water phase (*p* < 0.05; Pearson correlations *r* = −0.42, −0.37, and −0.44, respectively).

In contrast to the water phase, the majority of the major taxa level in the sediment correlated with NO_2_^−^ + NO_3_^−^ (Additional file 8: Table S3). *Acidobacteria*, *Chlorobi*, *Chloroflexi*, *Deferribacteres*, *Gemmatimonadetes*, *Nitrospirae*, *Planctomycetes*, and *Verrucomicrobia* all correlated positively with NO_2_^−^ + NO_3_^−^ (*r* = 0.49 to 0.82, *p* < 0.05). Groups that correlated negatively included *Bacteroidetes*, *Parcubacteria* (OD1), *Omnitrophica* (OP3), *Firmicutes*, *Lenthisphaerae*, and *Spirochaetae* (*r* = −0.44 to −0.63, *p* < 0.05). Several of these phyla also positively correlated with PO_4_^ 3−^ (data shown in Additional file 8: Table S3) except *Proteobacteria* that negatively correlated (*r* = −0.66, *p* < 0.01). Correlations with SO_4_^ 2−^ were all negative and included the phyla of *Actinobacteria*, *Latescibacteria* (WS3), and Candidate division TA06 (*r* = −0.50 to 0.68, *p* < 0.05).

The *Sulfurimonas*-like OTUs in the water phase had a significant positive correlation with PO_4_^ 3−^ (*p* < 0.01; *r* = 0.75) while these OTUs in the water phase and sediments had a weak negative correlation to NO_2_^−^ + NO_3_^−^ (*p* < 0.05; *r* = −0.28 and −0.55, respectively; Additional file 8: Table S3).

### Metagenomic analysis of metabolic potential in the sediments

Sequence assembly approaches of anoxic-to-oxic, intermediate-to-oxic, and oxic-to-anoxic sediment samples yielded on average 50,750 DNA coding sequences of which 6874 were annotated against the UniprotKB/Swiss-Prot database, giving ~14% of known and ~86% hypothetical genes. Similar values have been reported previously in global marine metagenomic sequencing of deep water [57]. Metagenomic gene counts have previously been observed to be a useful tool to estimate the importance of chemical processes in the environment [58].

The annotation against the KEGG database of metagenomic functional genes, also present in the metatranscriptome (see below), revealed nitrification (ammonia oxidation) presence in the oxic-to-anoxic, intermediate-to-oxic, and anoxic-to-oxic sediment (counts per million reads (CPM): 53 ± 29, 30 ± 8, and 198 ± 92, respectively, *n =* 2 for each treatment, SD = 1; Additional file 9: Data S2). Genes suggested to code for dissimilatory nitrate reduction were present in all samples (CPM 81 ± 47, 89 ± 70, and 137 ± 47) while sequences identified as assimilatory nitrate reduction genes were low in all samples (CPM 4 ± 6, 10 ± 14, and 68 ± 78). Genes for nitrogen fixation were only found in the anoxic-to-oxic sediment (CPM 0 ± 0, 0 ± 0, and 56 ± 79). In addition, sequences for genes attributed to dissimilatory sulfate reduction were more abundant in the anoxic-to-oxic sediment (CPM 242 ± 88, 226 ± 77, and 542 ± 11), while the amount of sequences for genes attributed to assimilatory sulfate reduction was highest in the intermediate-to-oxic sediment (CPM 263 ± 94, 809 ± 163, and 502 ± 148). Sequences for genes attributed to thiosulfate oxidation (coding for the Sox complex) was present in low abundance in all sediments (CPM 12 ± 6, 5 ± 7, 28 ± 32). Methane oxidation genes from formaldehyde assimilation were present in all treatments with the most sequences in the anoxic-to-oxic sediment (CPM 65 ± 12, 101 ± 31, and 228 ± 144).

### Metatranscriptomics of metabolic functions in the sediments

The de novo assembly of RNA sequences yielded 204,997 transcripts of which 57,386 could be annotated against the UniprotKB/Swiss-Prot (Additional file 9: Data S2) and PFAM reference databases (the two databases gave similar findings and therefore, only results from the UniprotKB/Swiss-Prot are presented hereafter). In the oxic sediments (oxic control: 4938 ± 0 and oxic-to-anoxic: 6272 ± 176 genes), fewer genes were expressed than in the intermediate-to-oxic/to-anoxic (7771 ± 375 and 7643 ± 303 genes, respectively) and anoxic control/to-oxic sediments (7859 ± 414 and 8274 ± 319 genes, respectively; Additional file 9: Data S2). RNA transcripts with significantly different FPKM counts according to edgeR analysis are presented in Additional file 9: Data S2 (UniprotKB/Swiss-Prot).

**Fig. 4:**
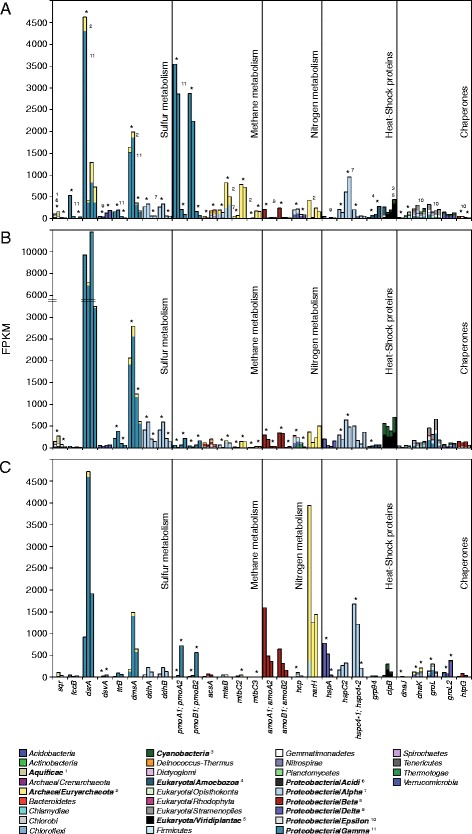
RNA transcripts of major metabolic processes in the sediments. Selected functional groups of RNA transcripts from the sediment after incubation in darkness for 21 days (RNA transcripts that were ≥100 FPKM in one of the treatments are shown). Functional groups were chosen based on statistical significance from edgeR analysis of the studied chemistry and 16S rRNA gene data. **a** Anoxic-to-oxic (*n* = 2, *two left bars* for each gene) and anoxic control (*n* = 2*, two right bars* for each gene). **b** Intermediate-to-oxic (*n* = 2, *left bars*) and intermediate-to-anoxic (*n* = 2, *right bars*). **c** Oxic-to-anoxic (*n* = 2, *left bars*) and oxic control (*n* = 1, *right bar*). *Stars* denote a fold change of at least 1.5 for the average of the two replicates compared to each replicate of the other shown treatment. All values show TMM normalized FPKM units. The *colors* denote relative abundance (% relative to column height) of taxonomic groups for the annotated RNA transcripts (i.e., derived from top hit organism in the UniprotKB/Swiss-Prot database)

Several of the most abundant known genes in the RNA transcripts were associated with inorganic sulfur transformations indicating activity of oxic and anoxic sulfur cycling in the different treatments (Fig. 4). RNA transcripts annotated for aerobic sulfide oxidation to sulfate were higher in the anoxic-to-oxic and intermediate-to-oxic cores compared to the anoxic controls or intermediate cores turned anoxic. These included genes for oxidizing sulfide to sulfur, such as sulfide:quinone oxidoreductase (*sqr*), sulfide dehydrogenase (*fccB*), and dissimilatory sulfite reductase (*dsrA*) that can act in reverse [59]. Spearman correlations of genes and chemistry data showed a significant negative correlation between *sqr* and sulfate concentration (*r* = 0.68, *p* < 0.05; Additional file 10: Table S4). In addition, heterodisulfide reductase (*hdrA*), used in both methanogenesis [60] and zero-valent sulfur oxidation [61], were present in all cores from the different treatments (Additional file 9: Data S2). Finally, RNA transcripts for sulfate adenylyltransferase (*sat*) that is suggested to be used in reverse to oxidize sulfite to sulfate [62] were also present in all cores from all treatments (Additional file 9: Data S2). In the UniprotKB/Swiss-Prot database, these RNA transcripts (Additional file 11: Data S3) were ascribed to *Acidithiobacillus/Aquifex* (*sqr*) and *Gammaproteobacteria* (*fccB* and *dsrA*). On the species level, *fccB* and *dsrA* were ascribed to the sulfide oxidizer *Allochromatium vinosum* (the *Chromatiaceae* family was present in the 16S rRNA gene data).

In the anoxic control, RNA transcripts annotated as sulfite reductase (*dsvA*) acting in anaerobic dissimilatory sulfate reduction were higher (FPKM 128 and 192 FPKM for each anoxic control replicate) compared to all other treatments that had FPKM values below 80. These transcripts were ascribed to the genus *Desulfovibrio* (e.g., species *Desulfovibrio vulgaris*) that was also present in the 16S rRNA gene dataset (Additional file 6: Data S1). Finally, tetrathionate reductase RNA transcript (*ttrB*) encoding anaerobic reduction of tetrathionate (S_4_O_6_^2−^) to thiosulfate (S_2_O_3_^2−^) [63] was higher in anoxic-to-oxic (156 to 198 FPKM) and intermediate-to-oxic (217 to 382 FPKM) compared to the anoxic control and intermediate-to-anoxic that had FPKM values below 60 and 110, respectively.

RNA transcripts coding for organic sulfur compound cycling by anaerobic dimethyl sulfoxide (DMSO) reductase (*dmsA*) that converts DMSO to dimethyl sulfide (DMS) were higher in the anoxic-to-oxic (1640 to 1993 FPKM) and intermediate-to-oxic treatments (2060 to 2795 FPKM) compared to the anoxic control and intermediate-to-anoxic with FPKM values below 400 and 1250 FPKM, respectively. In addition, transcript counts for the reverse process of anaerobic DMS oxidation back to DMSO by dimethyl sulfide dehydrogenase (*ddhAB*) were also higher under the same conditions (Additional file 9: Data S2). The genes *ttrB*, *dmsA*, and *ddhAB* all had a positive significant correlation with sulfate concentrations (*r* = 0.72–0.78, *p* < 0.05; Additional file 10: Table S4). The RNA transcripts for *dmsA* were affiliated with the class *Halobacteria* belonging to the *Euryarchaeota* phylum, while those for *ddhAB* were affiliated with the *Alphaproteobacteria* family *Rhodobacterales* (Additional file 11: Data S3). Both of these lineages were present in the 16S rRNA gene dataset (Additional file 6: Data S1).

RNA transcripts for genes mediating methane oxidation (*pmoA1A2* and *pmoB1B2* encoding methane monooxygenase) were higher in anoxic-to-oxic (2232 to 3542 FPKM) compared to the anoxic control (<220 FPKM) as well as in the oxic control (720 for *pmoA1A2*, and 564 for *pmoB1B2*; *n =* 1) compared to the oxic-to-anoxic treatment that had FPKM values below 40 (Fig. 4). In contrast, RNA transcripts for genes coding for methane oxidation were not higher in the intermediate-to-oxic treatments (Additional file 9: Data S2). RNA transcripts for *pmoA1A2* and *pmoB1B2* had a significant positive correlation with redox potential (i.e., high redox had more of these RNA transcripts; *r* = 0.83, *p* < 0.01; Additional file 10: Table S4) and were ascribed to the *Gammaproteobacteria* reference organism *Methylococcus capsulatus* (Additional file 11: Data S3) that was present in the 16S rRNA gene dataset (Additional file 6: Data S1). RNA transcripts coding for methanogenesis (*acsA*, *mtaB*, and *mtbC2C3*) were higher in the anoxic control (168 to 821 FPKM) compared to the anoxic-to-oxic sediment (13 to 194 FPKM). Also, *mtaB* and *mtbC2C3* had more RNA transcripts attributed in the intermediate-to-anoxic cores (33 to 206 FPKM) when compared intermediate-to-oxic (2 to 72 FPKM; Fig. 4). The *acsA* RNA transcripts were affiliated with a wide diversity of bacteria including, e.g., *Alpha-*, *Beta-*, *Epsilon-*, and *Gammaproteobacteria* (Additional file 11: Data S3). RNA transcripts for *mtaB* affiliated with the archaeal class *Methanomicrobia* and *Alphaproteobacteria* class *Rickettsiales*, while *mtbC2C3* affiliated only with *Methanomicrobia* (Additional file 11: Data S3); both of these classes were present in the 16S rRNA gene dataset (Additional file 6: Data S1). RNA transcripts encoding *hdrA* used during anaerobic methanogenesis [64] were present in all cores for all treatments.

RNA transcripts coding for nitrate reductase (*narH*) were higher in the oxic-to-anoxic cores (1250 to 394 FPKM) compared the oxic control (1436 FPKM). In addition, RNA transcripts coding for *narH* were found at lower amounts in the other treatments (Fig. 4). RNA transcripts attributed to *narH* also correlated positively with NO_2_^−^ + NO_3_^−^ (*r* = 0.68, *p* < 0.05; Additional file 10: Table S4). These RNA transcripts were mainly affiliated with halophilic denitrifying archaeal species belonging to the class *Halobacteria* (closest available affiliation in UniProt KB: *Haloferax mediterranei*; Additional file 11: Data S3). *Halobacteria* were also present in the 16S rRNA gene data (Additional file 6: Data S1) and phylogenetic analysis, with reference sequences of Marine group I, II, III, IV, and *Haloferax* species, indicated that the OTUs aligned with the order *Halobacteriales* and the ‘Deep Sea Hydrothermal Vent Group 6’ (DHVEG-6; Additional file 12: Figure S4). These *Halobacteria* in the sediment might thrive in micro-niches or contain low-saline species as has been reported previously in sulfur and sulfide rich conditions [65].

In general, there was a low level of RNA transcripts coding for the general stress response in all treatments (Fig. 4). One exception was for the HSP70 family (*hspA*) that had higher RNA transcripts in the oxic-to-anoxic cores (538 to 778 FPKM) compared to the oxic control (46 FPKM). A second exception was for HSP90 family genes (*hspC2/4–1/4–2*) that had more RNA transcripts attributed in the oxic-to-anoxic cores (1216 to 1682 FPKM) compared to the oxic control (205 FPKM). The taxonomic affiliations of RNA transcripts coding for general stress were diverse and also included eukaryotes such as *Amoebozoa*. In addition, the chaperones *dnaK* and *htpG* were assigned to *Sulfurimonas* and *Sulfurovum* spp. (Additional file 11: Data S3) that tended to be higher in treatments turned oxic or the oxic control cores.

## Discussion

In this study, we investigated changes in the microbial community composition and metabolic function in long-term oxic and anoxic sediments plus intermediate sediments from a Baltic Sea coastal bay. The tightly clustered microbial communities in the oxic sediment field sample, control cores, and oxic-to-anoxic incubations were likely due to long time exposure to oxygen selecting for obligate aerobes (PCA, Fig. 2). In contrast, microbial communities in the anoxic-to-oxic sediment incubations were slightly less clustered, potentially as facultative anaerobes could adapt to increased oxygen concentrations. The intermediate sediment communities were most widespread compared to other locations, with populations of generalists adapted to the changing oxygen concentration. This suggested that historical oxygen concentrations will affect the microbial response to shifts in oxygen concentrations.

Reduction of sulfate to H_2_S by sulfate-reducing bacteria is prevalent in Baltic Sea sediments including anoxic sediment rich in labile organic matter [66] and the generated sulfide can be re-oxidized to sulfate in aerobic conditions [67]. Recent molecular phylogenetic and genetic analyses show that many genes coding for sulfur cycling processes occur in anoxic environments. For example, a metagenomic investigation of the waters in the oxygen-minimum zone outside the northern Chilean coast found that genes for both sulfate reduction and sulfide oxidation were present in the anoxic water [68]. Furthermore, with the use of isotope-labeled sulfate and oxygen, cryptic sulfur cycling was observed in anoxic salt march sediments. The results indicate microbial reduction of sulfate to H_2_S followed by microbial re-oxidation of H_2_S into intermediary sulfur species and eventually sulfate [69]. Consistent with this data, the anoxic sediment turned oxic compared to cores kept or turned anoxic had increased sulfate in the pore-water (Fig. 1); more RNA transcript numbers for inorganic sulfur oxidation genes (Fig. 4) [62, 70]; and an increase in *Sulfurovum*-like OTUs (Fig. 3) that contain genes attributed to sulfide oxidation to sulfate [71, 72] suggesting they carried out sulfur oxidation. Considering that sulfur cycling RNA transcripts could not be directly linked to *Sulfurovum* and instead transcripts attributed to chaperones *dnaK* and *htpG* were linked to this genus (Additional file 11: Data S3), it is possible that the sulfur cycling activity by *Sulfurovum* had declined after 21 days of incubation. Also consistent with these findings was that compared to the field data *Sulfurimonas*-like populations increased in cores maintained anoxic, while negatively correlating with the NO_2_^−^ + NO_3_^−^ concentration (Additional file 8: Table S3) suggesting they oxidized sulfide coupled to nitrate reduction. However, RNA transcripts coding for nitrate reductase (*narH*) mainly aligned with *Euryarchaeota*. It is possible that a precise taxonomic affiliation cannot be determined for this conserved gene, and it can only be concluded that the microbial community utilized NO_3_^−^ as a terminal electron acceptor under anaerobic conditions [73]. Taken together, the RNA transcripts coding for sulfur oxidation and reduction reactions and the low concentrations of zero-valent sulfur and reduced sulfur compounds throughout the incubations (Additional file 3: Table S2) suggested that a cryptic cycling of sulfur and sulfur compounds occurred in the sediment. Furthermore, we observed genes used in anaerobic metabolism in the 1 cm sediment slices turned oxic. This indicated that changes in oxygen conditions in the sediment surface oxic layer (reaching ~1.5 mm) affected anaerobic microbial metabolism in the underlying anoxic layer.

As described above, the sulfur cycling related 16S rRNA gene amplicons, mRNA transcripts, and chemistry data in conjunction with qualitative observations that the sediment surface turned brown and had a lowered sulfide odor strongly supported that *Sulfurovum*-like populations were favored in the surface of anoxic-to-oxic incubations. This is consistent with the description of this genus that grows via sulfur compound oxidation with the reduction of oxygen or nitrate [74]. These populations would potentially act as a H_2_S oxidation barrier between the oxygenated sediment surface and the deeper anoxic H_2_S rich sediment and would accelerate removal of H_2_S.

Most methane oxidation occurs in the sediment surface and limits the amount of this potent greenhouse gas to escape from the water column [75]. RNA transcripts coding for methane oxidation were highest in the anoxic-to-oxic and oxic control (Fig. 4). This was likely explained by oxygen reaching anoxic layers below the sediment surface that triggered transcription of these genes. The RNA transcripts encoding *pmoAB* and 16S rRNA data suggested methane oxidation in the anoxic-to-oxic and oxic control was mediated by *Methylococcales*, a microorganism that typically grows in the upper layers of marine sediment [76]. In contrast, methanotrophy-related RNA transcripts were lower in the intermediate-to-oxic sediment compared to the intermediate-to-anoxic. Thus, stored methane had potentially been metabolized during episodic oxygenation events or alternatively, the methanogenic archaea were inhibited during these oxygenation episodes. Irrespective of the precise mechanism, intermediate zones with fluctuating oxygen concentrations may not produce the greenhouse gas methane while an anoxic-to-oxic shift resulted in increased methanotrophy that would consume the methane and halt its escape to the atmosphere. Thus, gene expression analysis indicated an intricate balance between production and consumption of methane in intermittently and permanently anoxic sediments.

It has previously been shown that degradation of experimentally added organic matter to sediments is slower under anoxic compared to oxic conditions [22, 23]. In addition, Hulthe G, Hulth S, and Hall POJ [77] found that old, buried organic matter was more rapidly oxidized upon re-oxygenation of anoxic sediments compared to fresh organic matter in the sediment surface (which was degraded at similar rates both aerobically and anaerobically). A statistically significant decrease in organic matter was observed when the anoxic sediment was turned oxic, but not in the initially anoxic intermediate sediment upon oxygenation (Table 1). Considering that more organic matter is preserved in low oxygen zones [21], here we have shown that the preserved organic matter in the long-term studied anoxic zone was degraded upon oxygenation. In contrast, this was not the case for the intermediate area subjected to episodic variations in oxygen concentration where the stored organic matter would likely have been periodically depleted.

## Conclusions

**Fig. 5:**
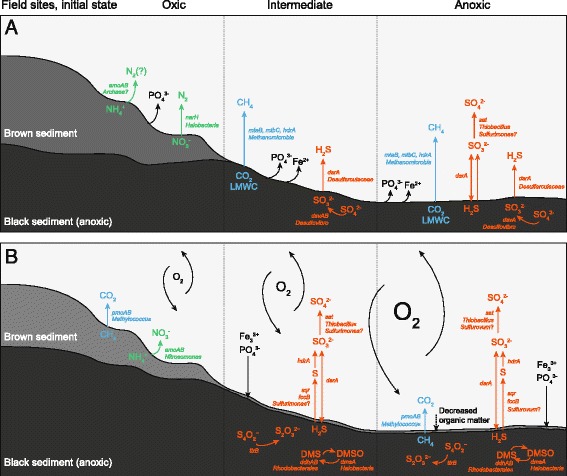
Conceptual model of the bay during transitions in oxygen concentration. The model shows major changes/findings from the 16S rRNA gene, mRNA transcripts, and chemistry data after 21 days of incubation. The sulfur cycle is shown in *orange*, methane as *light blue*, and nitrogen as *green*. **a** denotes initial conditions turned/maintained anoxic, while **b** denotes initial conditions turned/maintained oxic. *LMWC* low molecular weight carbon substrates

In conclusion, we show for the first time the large changes in microbial populations and genes suggested to code for metabolic processes inflicted by shifts in oxygen levels in coastal oxic, intermediate, and anoxic zones (Fig. 5). In a coastal anoxia dead zone remediation scenario, oxygenation of the sediment surface was followed by an increase in bacterial populations affiliated with the genus *Sulfurovum* and RNA transcripts attributed to sulfur cycling (such as oxidation of toxic H_2_S) that would potentially prevent H_2_S from ascending into the water column. Additionally, a decrease in methane release was indicated by higher amounts of RNA transcripts attributed to methane oxidation. This was also indicated by lower amounts of RNA transcripts for methanogenesis when compared to the anoxic controls. Oxygenation of anoxic sediment would therefore likely limit the release of this greenhouse gas to the atmosphere. These findings have important implications in the context of proposed remediation strategies for coastal dead zones, such as those found in the Baltic Sea. These strategies include mixing the water column using pump stations, circulating oxygenated water to the sea bottom [78, 79]. One such study was conducted in a fjord on the west coast of Sweden where the re-oxygenated bottom zone showed a decrease in phosphate [80] that would help hypoxic and anoxic coastal zones to recover from eutrophication [81]. Addition of oxygen in conjunction with microbial removal of H_2_S would help macro-organism communities to re-establish in the surface sediment and benthic zone that was supported by increased hatching of zooplankton eggs in a similar experiment [29]. Additionally, re-oxygenation of the anoxic sediment surface resulted in decreased stored organic matter (Table 1) and it was estimated that it would take approximately 73 days for the anoxic sediment to reach the level of organic matter in the long-term oxic field sediment (without further addition of organic matter from the water column that would likely be oxidized first). These ~2.5 months may be a crucial time necessary for restoration of coastal dead zones and would potentially require continuous re-oxygenation. Based on the results found in this study, we suggest that remediation efforts of dead zones effectively depend on the microbial community response and that these responses will vary locally/regionally depending on anoxia history. Furthermore, it was shown that re-oxygenation efforts to remediate dead zones could ultimately be facilitated by in situ microbial molecular mechanisms involved in removal of toxic H_2_S and the potent greenhouse gas methane.

## Additional files

Additional file 1: **Text S1.** Detailed methodology of field sampling and the sub-sampling procedure. (DOCX 271 kb)

Additional file 2: Table S1. A full list of reads for the various sequencing programs including before and after merging reads, quality trimming and bioinformatic statistics on the assemblies constructed from the metagenomes and de-novo assembled metatranscriptoms. (DOCX 62 kb)

Additional file 3: Table S2. Chemistry data from the water phase and the sediment collected in the field, during, and at the end of the incubation experiment. All values are averages of triplicates (SD = 1) except oxic and anoxic controls (*n* = 2), water and sediment oxic-to-anoxic, and sediment anoxic-to-oxic (both *n* = 2). All chemistry data in the sediment was measured using the pore-water except organic matter which was measured using the loss on ignition method. (XLSX 16 kb)

Additional file 4: Figure S1. Rarefraction curves of counts and OTUs for each treatment in the water phase and sediment (counts were subsampled to lowest sample size). Each curve denotes a biological replicate. (PDF 104 kb)

Additional file 5: Figure S2. Phylogenetic tree showing OTUs derived from the 16S rRNA gene analysis. Included OTUs had a relative abundance higher than 1% in one or more samples derived from the top 1-cm sediment surface sliced in the field and at the end of the incubation experiment. Sequences downloaded from NCBI GenBank have been marked with a *dashed line* in the heat map. The tree was rooted with a marine *Actinomycete* (NCBI accession: AJ866956.1). (PDF 456 kb)

Additional file 6: **Data S1.** All OTUs and a separate list for the top 10 abundant, annotated against the SILVA database. All values are reported as relative abundances, with triplicates (SD = 1) except water and sediment oxic-to-anoxic and sediment anoxic-to-oxic (both *n* = 2). The second sheet shows the taxonomy results based on extracted 16S and 18S rRNA gene sequences from the sediment metagenome dataset. (XLSX 1447 kb)

Additional file 7: Figure S3. 16S rRNA gene sequence data of annotated OTUs in the sediment of an additional oxygen transition experiment. Ten sediment cores from the anoxic site were sampled on the 15 May 2014. During 26 days of incubation, 5 cores were maintained anoxic while the other 5 cores were turned oxic using similar methodology as describe in the main article. The *Proteobacteria* have been divided into classes, and the *Epsilonproteobacteria* has been divided into two genus *Sulfurimonas* and *Sulfurovum*, and other *Epsilonproteobacteria*. (PDF 151 kb)

Additional file 8: Table S3. Pearson correlations of chemistry data and relative abundance of annotated phyla, classes, orders, and the genera *Sulfurimonas* and *Sulfurovum* in the water phase and the sediment. The *orange* shading denotes statistically significant *p* values at <0.01 (**) and <0.05 (*). The last table shows Spearman correlations of selected genes derived from statistically significant RNA transcripts (as shown in Fig. 4) and sediment chemistry data after 21 days of incubation. (DOCX 159 kb)

Additional file 9: **Data S2.** The list shows all results (FPKM), with a following separate list of the significant results, from the UniprotKB/Swiss-Prot database annotation of the de novo metatranscriptomic assemblies (*n* = 2, for each treatment except oxic control: *n* = 1), SD = 1. The second sheet shows the results from annotation of functional genes derived from the metagenome. (XLSX 2747 kb)

Additional file 10: Table S4. Spearman correlations of statistically significant genes from edgeR (as seen in Fig. 4) derived from the extracted sediment RNA and sediment chemistry measurements after 21 days of incubation. (DOCX 50 kb)

Additional file 11: **Data S3.** Reference organisms from the UniProtKB/Swiss-Prot database tied to the annotation of chosen ‘RNA transcript-related genes’ (top five hits are shown). (XLSX 114 kb)

Additional file 12: Figure S4. Phylogenetic unrooted maximum-likelihood tree (bootstrapped 100 times) showing archaeal OTUs derived from the 16S rRNA gene analysis compared to reference sequences downloaded from NCBI GenBank that are colored *red*. The *scale bar* represents nucleotide substitutions per site (i.e., substitutions divided by sequence length). (PDF 244 kb)
