# Supplementary material for: Shifts in coastal sediment oxygenation cause pronounced changes in microbial community composition and associated metabolism
Source: Microbiome. 2017 Aug 9;5:96. doi: 10.1186/s40168-017-0311-5 (PMC5549381; doi:10.1186/s40168-017-0311-5)
Supplement: Supplementary file 1 — Text S1. Detailed methodology of field sampling and the sub-sampling procedure. (DOCX 271 kb) [file 40168_2017_311_MOESM1_ESM.docx]

**Sampling and handling of sediment cores**

The studied bay was chosen as it contained sampling sites with different oxygenation histories based upon previous dissolved O_2_ measurements showing hypoxia (< 2 mg/L O_2_) levels from various sites in the area. Hypoxia was observed to occur during all months (with some sites being episodic/seasonally hypoxic mainly during autumn) from 1995 to 2015. Data is available by the Kalmar county coastal water committee at (Swedish website):

<http://www.kalmarlanskustvatten.org/index.php?option=com_herbfileselector&view=herbfileselector&Itemid=3>. We also conducted a pilot study in October 2013 wherein we sampled sediment cores from the same sites as this study and observed the anoxic site to have black sediment with a strong odor of H_2_S (see table below for O_2_ measurements; dissolved O_2_ in the bottom was not measured at the oxic site at that time, but the sediment was observed to be light brown).

The table shows measurements from this study (November 2013) of dissolved O_2_ (mg/L), water depth, salinity and temperature from the different field sites. Measurements of O_2_ were conducted in the bottom water ~20 cm overlying the sediment. Additional O_2_ data is shown from sampling trips conducted in October 2013, May 2014, October 2014 and April 2015).

|  | Oxic region | Intermediate region | Anoxic region |
| --- | --- | --- | --- |
| WGS 84 Coordinates (Lat., Lon.) | 57 53.214, 16 35.934 | 57 53.545, 16 35.476 | 57 53.531, 16 35.165 |
| Water depth (m) | 6.5 | 21 | 31.2 |
| Salinity (‰) | 6.2 | 6.6 | 6.7 |
| Bottom temp. (°C) | 6.5 | 2.8 | 2.9 |
| O_2_ Oct 16^th^ 2013 |  | 5.49 | 0.62 |
| O_2_ Nov 16^th^ 2013 | 11.3 | 0.8 | 0.85 |
| O_2_ May 14^th^ 2014* |  |  | 0.2 |
| O_2_ Oct 9^th^ 2014 | 10.1 |  |  |
| O_2_ Apr 16^th^ 2015 | 10.6 |  | ** |

* Broman, E., Brüsin, M., Dopson, M., and Hylander, S. (2015). Oxygenation of anoxic sediments triggers hatching of zooplankton eggs. Proceedings of the Royal Society of London B: Biological Sciences 282(1817). doi: 10.1098/rspb.2015.2025.

** Dissolved O_2_ was not measured but the sediment was observed to be black with a strong odor of H_2_S.


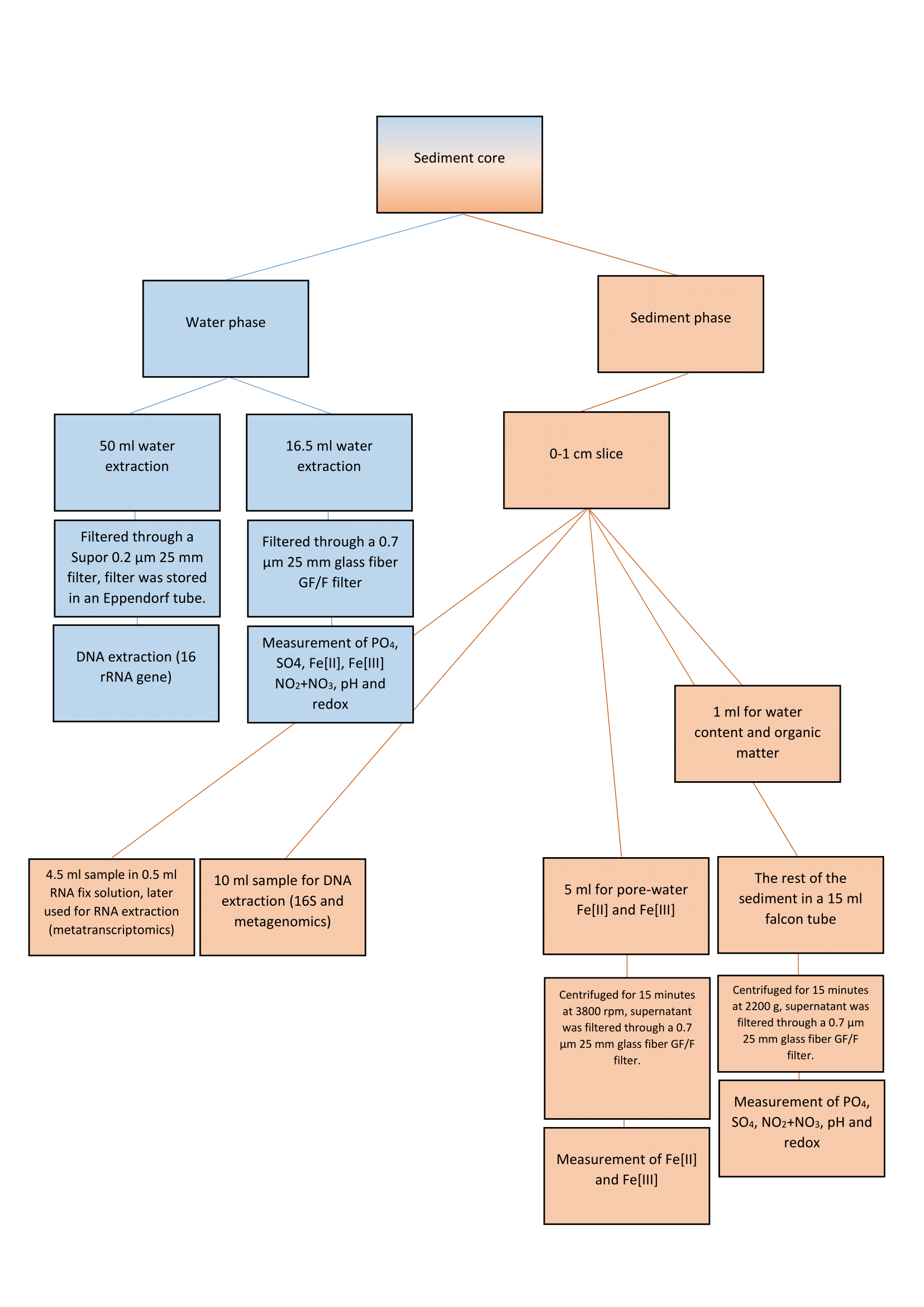
A schematic overview of the sub-sampling procedure conducted every 4^th^ day in the water phase overlying the sediment. Sediment was sliced in the field and at the end of the incubation experiment after 21 days.
