# Supplementary figures and images for: Shifts in coastal sediment oxygenation cause pronounced changes in microbial community composition and associated metabolism
Source: Microbiome. 2017 Aug 9;5:96. doi: 10.1186/s40168-017-0311-5 (PMC5549381; doi:10.1186/s40168-017-0311-5)

## Sediment

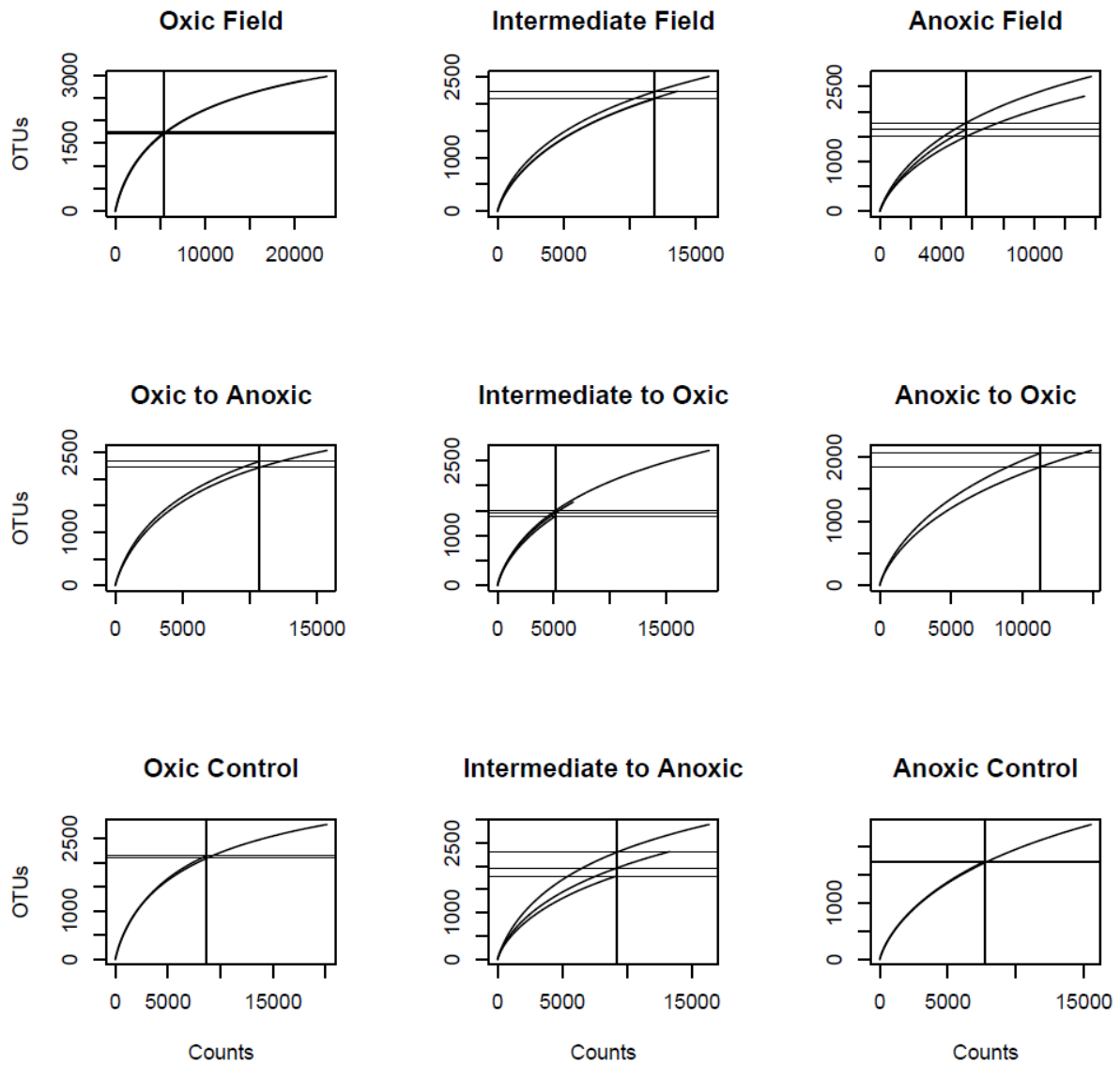

11

12

## Water

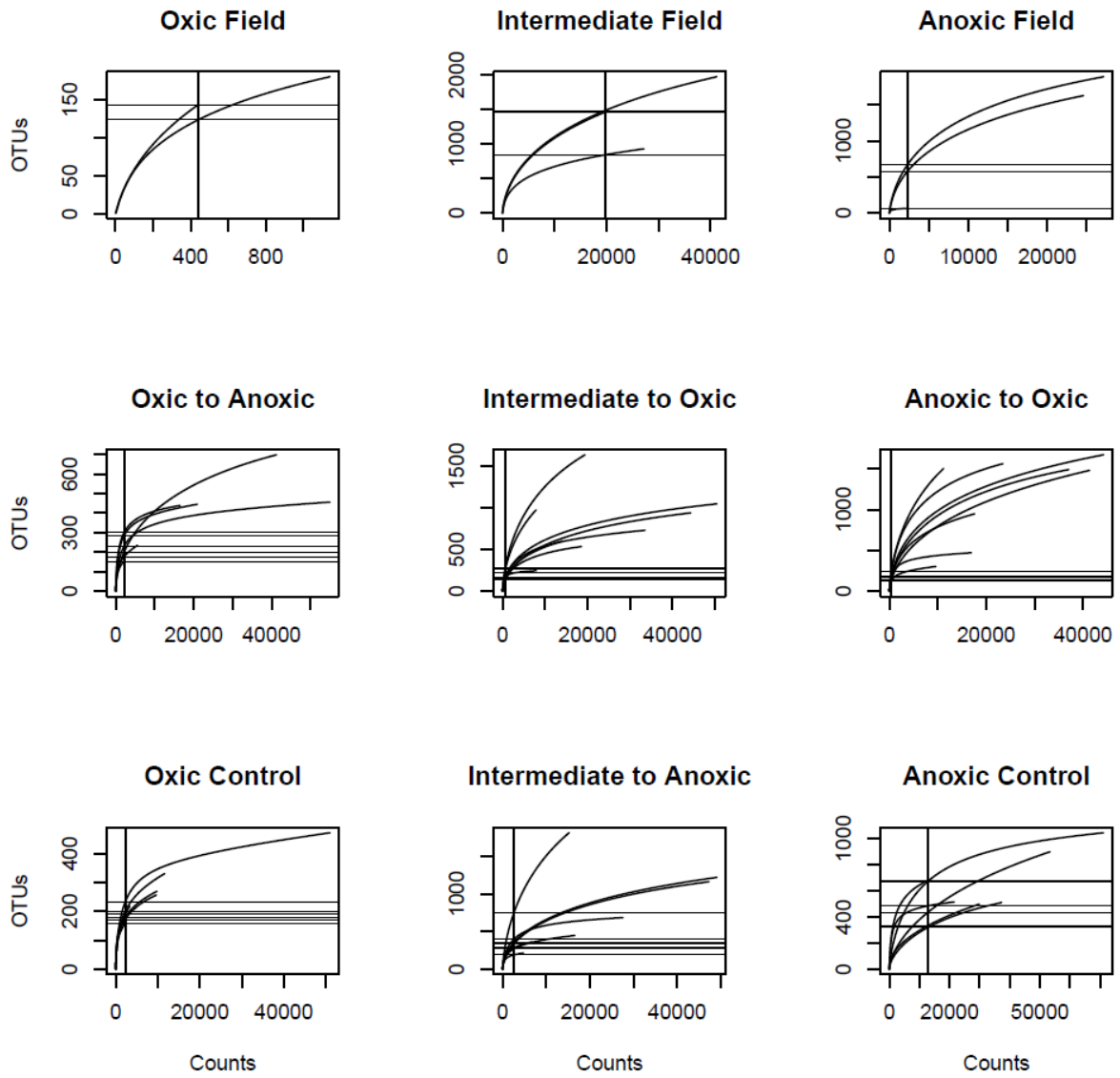

Supplement: Supplementary file 4 — Rarefraction curves of counts and OTUs for each treatment in the water phase and sediment (counts were subsampled to lowest sample size). Each curve denotes a biological replicate. (PDF 104 kb) [file 40168_2017_311_MOESM4_ESM.pdf]

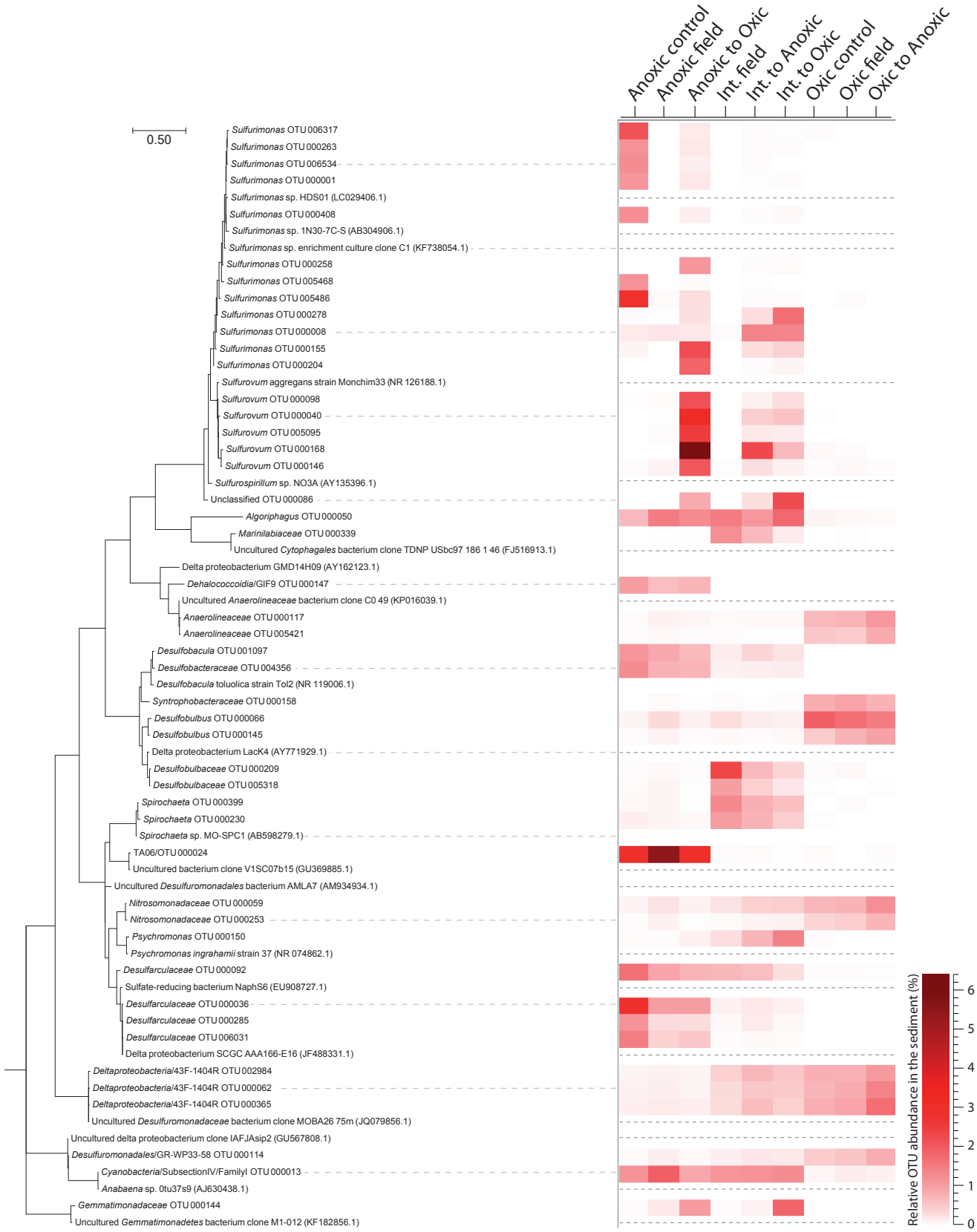

Supplement: Supplementary file 5 — Phylogenetic tree showing OTUs derived from the 16S rRNA gene analysis. Included OTUs had a relative abundance higher than 1% in one or more samples derived from the top 1-cm sediment surface sliced in the field and at the end of the incubation experiment. Sequences downloaded from NCBI GenBank have been marked with a dashed line in the heat map. The tree was rooted with a marine Actinomycete (NCBI accession: AJ866956.1). (PDF 456 kb) [file 40168_2017_311_MOESM5_ESM.pdf]

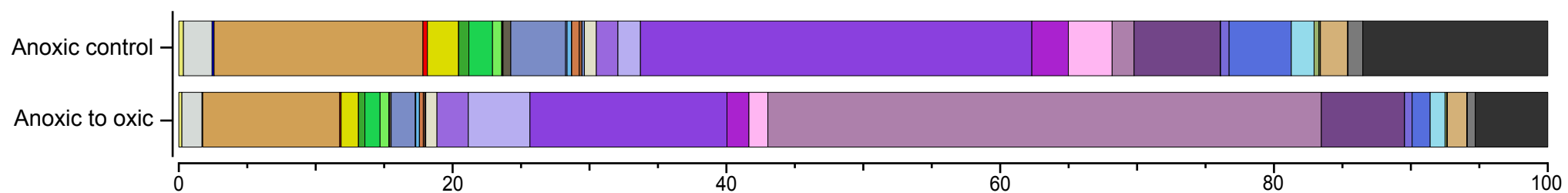

Supplement: Supplementary file 7 — 16S rRNA gene sequence data of annotated OTUs in the sediment of an additional oxygen transition experiment. Ten sediment cores from the anoxic site were sampled on the 15 May 2014. During 26 days of incubation, 5 cores were maintained anoxic while the other 5 cores were turned oxic using similar methodology as describe in the main article. The Proteobacteria have been divided into classes, and the Epsilonproteobacteria has been divided into two genus Sulfurimonas and Sulfurovum, and other Epsilonproteobacteria. (PDF 151 kb) [file 40168_2017_311_MOESM7_ESM.pdf]
