# Supplementary material for: Shifts in coastal sediment oxygenation cause pronounced changes in microbial community composition and associated metabolism
Source: Microbiome. 2017 Aug 9;5:96. doi: 10.1186/s40168-017-0311-5 (PMC5549381; doi:10.1186/s40168-017-0311-5)
Supplement: Supplementary file 8 — Pearson correlations of chemistry data and relative abundance of annotated phyla, classes, orders, and the genera Sulfurimonas and Sulfurovum in the water phase and the sediment. The orange shading denotes statistically significant p values at <0.01 (**) and <0.05 (*). The last table shows Spearman correlations of selected genes derived from statistically significant RNA transcripts (as shown in Fig. 4) and sediment chemistry data after 21 days of incubation. (DOCX 159 kb) [file 40168_2017_311_MOESM8_ESM.docx]

Spearman correlations of statistically significant genes from edgeR (as seen in figure 4) derived from the extracted sediment RNA and sediment chemistry measurements after 21 days of incubation.
