# Supplementary material for: Shifts in coastal sediment oxygenation cause pronounced changes in microbial community composition and associated metabolism
Source: Microbiome. 2017 Aug 9;5:96. doi: 10.1186/s40168-017-0311-5 (PMC5549381; doi:10.1186/s40168-017-0311-5)
Supplement: Supplementary file 12 — Phylogenetic unrooted maximum-likelihood tree (bootstrapped 100 times) showing archaeal OTUs derived from the 16S rRNA gene analysis compared to reference sequences downloaded from NCBI GenBank that are colored red. The scale bar represents nucleotide substitutions per site (i.e., substitutions divided by sequence length). (PDF 244 kb) [file 40168_2017_311_MOESM12_ESM.pdf]

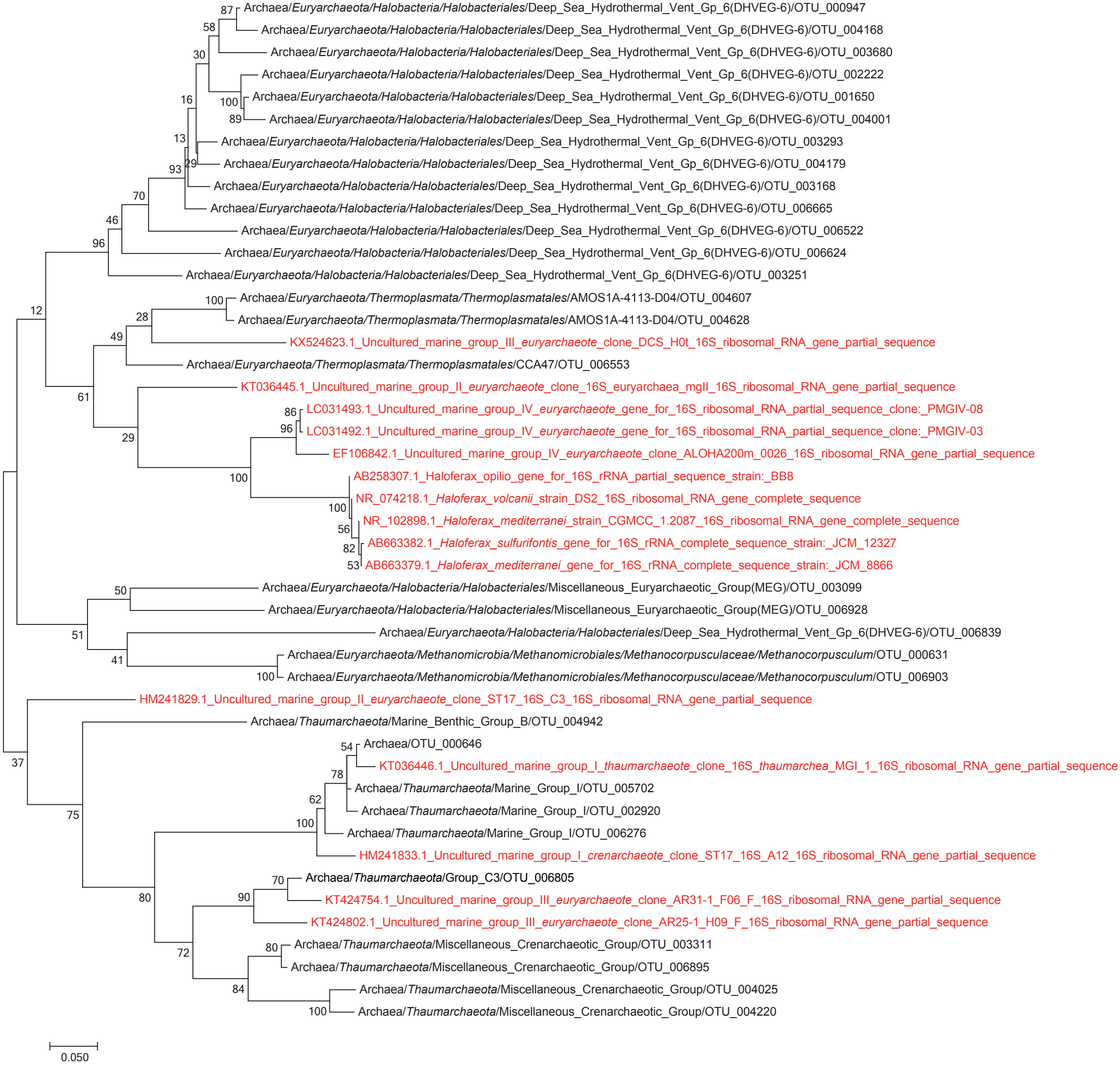

Archaea/Euryarchaeota/Halobacteria/Halobacteriales/Deep_Sea_Hydrothermal_Vent_Gp_6(DHVEG-6)/OTU_000947
Archaea/Euryarchaeota/Halobacteria/Halobacteriales/Deep_Sea_Hydrothermal_Vent_Gp_6(DHVEG-6)/OTU_004168
Archaea/Euryarchaeota/Halobacteria/Halobacteriales/Deep_Sea_Hydrothermal_Vent_Gp_6(DHVEG-6)/OTU_003680
Archaea/Euryarchaeota/Halobacteria/Halobacteriales/Deep_Sea_Hydrothermal_Vent_Gp_6(DHVEG-6)/OTU_002222
Archaea/Euryarchaeota/Halobacteria/Halobacteriales/Deep_Sea_Hydrothermal_Vent_Gp_6(DHVEG-6)/OTU_001650
Archaea/Euryarchaeota/Halobacteria/Halobacteriales/Deep_Sea_Hydrothermal_Vent_Gp_6(DHVEG-6)/OTU_004001
Archaea/Euryarchaeota/Halobacteria/Halobacteriales/Deep_Sea_Hydrothermal_Vent_Gp_6(DHVEG-6)/OTU_003293
Archaea/Euryarchaeota/Halobacteria/Halobacteriales/Deep_Sea_Hydrothermal_Vent_Gp_6(DHVEG-6)/OTU_004179
Archaea/Euryarchaeota/Halobacteria/Halobacteriales/Deep_Sea_Hydrothermal_Vent_Gp_6(DHVEG-6)/OTU_003168
Archaea/Euryarchaeota/Halobacteria/Halobacteriales/Deep_Sea_Hydrothermal_Vent_Gp_6(DHVEG-6)/OTU_006665
Archaea/Euryarchaeota/Halobacteria/Halobacteriales/Deep_Sea_Hydrothermal_Vent_Gp_6(DHVEG-6)/OTU_006522
Archaea/Euryarchaeota/Halobacteria/Halobacteriales/Deep_Sea_Hydrothermal_Vent_Gp_6(DHVEG-6)/OTU_006624
Archaea/Euryarchaeota/Halobacteria/Halobacteriales/Deep_Sea_Hydrothermal_Vent_Gp_6(DHVEG-6)/OTU_003251
Archaea/Euryarchaeota/Thermoplasmata/Thermoplasmatales/AMOS1A-4113-D04/OTU_004607
Archaea/Euryarchaeota/Thermoplasmata/Thermoplasmatales/AMOS1A-4113-D04/OTU_004628
KX524623.1_Uncultured_marine_group_III_euryarchaeote_clone_DCS_H0t_16S_ribosomal_RNA_gene_partial_sequence
Archaea/Euryarchaeota/Thermoplasmata/Thermoplasmatales/CCA47/OTU_006553
KT036445.1_Uncultured_marine_group_II_euryarchaeote_clone_16S_euryarchaea_mgII_16S_ribosomal_RNA_gene_partial_sequence
LC031493.1_Uncultured_marine_group_IV_euryarchaeote_gene_for_16S_ribosomal_RNA_partial_sequence_clone:_PMGIV-08
LC031492.1_Uncultured_marine_group_IV_euryarchaeote_gene_for_16S_ribosomal_RNA_partial_sequence_clone:_PMGIV-03
EF106842.1_Uncultured_marine_group_IV_euryarchaeote_clone_ALOHA200m_0026_16S_ribosomal_RNA_gene_partial_sequence
AB258307.1_Haloferax_opilio_gene_for_16S_rRNA_partial_sequence_strain:_BB8
NR_074218.1_Haloferax_volcanii_strain_DS2_16S_ribosomal_RNA_gene_complete_sequence
NR_102898.1_Haloferax_mediterranei_strain_CGMCC_1.2087_16S_ribosomal_RNA_gene_complete_sequence
AB663382.1_Haloferax_sulfurifontis_gene_for_16S_rRNA_complete_sequence_strain:_JCM_12327
AB663379.1_Haloferax_mediterranei_gene_for_16S_rRNA_complete_sequence_strain:_JCM_8866
Archaea/Euryarchaeota/Halobacteria/Halobacteriales/Miscellaneous_Euryarchaeotic_Group(MEG)/OTU_003099
Archaea/Euryarchaeota/Halobacteria/Halobacteriales/Miscellaneous_Euryarchaeotic_Group(MEG)/OTU_006928
Archaea/Euryarchaeota/Halobacteria/Halobacteriales/Deep_Sea_Hydrothermal_Vent_Gp_6(DHVEG-6)/OTU_006839
Archaea/Euryarchaeota/Methanomicrobia/Methanomicrobiales/Methanocorpusculaceae/Methanocorpusculum/OTU_000631
Archaea/Euryarchaeota/Methanomicrobia/Methanomicrobiales/Methanocorpusculaceae/Methanocorpusculum/OTU_006903
HM241829.1_Uncultured_marine_group_II_euryarchaeote_clone_ST17_16S_C3_16S_ribosomal_RNA_gene_partial_sequence
Archaea/Thaumarchaeota/Marine_Benthic_Group_B/OTU_004942
Archaea/OTU_000646
KT036446.1_Uncultured_marine_group_I_thaumarchaeote_clone_16S_thaumarchea_MGI_1_16S_ribosomal_RNA_gene_partial_sequence
Archaea/Thaumarchaeota/Marine_Group_I/OTU_005702
Archaea/Thaumarchaeota/Marine_Group_I/OTU_002920
Archaea/Thaumarchaeota/Marine_Group_I/OTU_006276
HM241833.1_Uncultured_marine_group_I_crenarchaeote_clone_ST17_16S_A12_16S_ribosomal_RNA_gene_partial_sequence
Archaea/Thaumarchaeota/Group_C3/OTU_006805
KT424754.1_Uncultured_marine_group_III_euryarchaeote_clone_AR31-1_F06_F_16S_ribosomal_RNA_gene_partial_sequence
KT424802.1_Uncultured_marine_group_III_euryarchaeote_clone_AR25-1_H09_F_16S_ribosomal_RNA_gene_partial_sequence
Archaea/Thaumarchaeota/Miscellaneous_Crenarchaeotic_Group/OTU_003311
Archaea/Thaumarchaeota/Miscellaneous_Crenarchaeotic_Group/OTU_006895
Archaea/Thaumarchaeota/Miscellaneous_Crenarchaeotic_Group/OTU_004025
Archaea/Thaumarchaeota/Miscellaneous_Crenarchaeotic_Group/OTU_004220
0.050
