# Supplementary material for: Shifts in coastal sediment oxygenation cause pronounced changes in microbial community composition and associated metabolism
Source: Microbiome. 2017 Aug 9;5:96. doi: 10.1186/s40168-017-0311-5 (PMC5549381; doi:10.1186/s40168-017-0311-5)
Supplement: Supplementary file 7 [file 40168_2017_311_MOESM10_ESM.docx]

gene	Phosphate µM	Nitrite + Nitrate µM	Sulfate mM	Total iron µM	pH	Redox mV	Organic matter %	Tetrathionate µM
sqr	** -0.86	-0.01	* 0.68	0.10	-0.54	0.37	-0.02	-0.22
fccB	-0.15	-0.04	0.42	* -0.71	-0.31	0.03	-0.41	0.32
dsrA	-0.42	0.09	0.50	0.33	-0.60	-0.31	0.27	0.03
sat	-0.45	-0.20	0.50	0.36	-0.49	-0.54	0.49	-0.25
dsvA	0.36	* -0.68	-0.63	-0.01	** 0.94	0.03	0.54	-0.18
dsvB	0.09	** -0.74	-0.37	0.31	* 0.89	-0.20	0.48	-0.24
ttrB	** -0.85	-0.15	* 0.72	0.10	-0.71	0.09	0.17	-0.14
dmsA	** -0.91	0.00	* 0.78	-0.02	-0.71	0.09	0.05	-0.13
ddhA	** -0.85	-0.02	* 0.78	0.07	-0.71	0.09	0.05	-0.09
ddhB	** -0.85	-0.02	* 0.78	0.07	-0.71	0.09	0.05	-0.09
pmoA1; pmoA2	-0.06	-0.40	0.18	-0.21	0.37	* 0.83	0.03	0.15
pmoB1; pmoB2	-0.06	-0.40	0.18	-0.21	0.37	* 0.83	0.03	0.15
hdrA	-0.44	-0.19	0.57	0.38	-0.66	-0.37	0.47	-0.18
hdrA2	-0.45	0.00	0.52	0.29	-0.60	-0.31	0.36	-0.02
acsA	0.16	-0.44	-0.35	0.40	0.31	-0.43	** 0.83	-0.24
mtaB	0.17	** -0.78	-0.38	0.19	** 0.94	-0.03	* 0.61	-0.26
mtbC2	0.42	* -0.72	-0.58	0.43	* 0.83	-0.09	0.52	-0.26
mtbC3	0.42	* -0.72	-0.58	0.43	* 0.83	-0.09	0.52	-0.26
amoA1; amoA2	-0.07	** 0.85	0.45	-0.31	* -0.83	-0.49	-0.58	0.53
amoB1; amoB2	-0.31	* 0.73	0.43	-0.40	-0.49	-0.26	-0.35	0.49
hcp	** -0.85	-0.34	0.57	0.05	-0.31	-0.20	0.33	-0.22
narH	0.36	* 0.68	0.32	-0.29	-0.54	-0.26	-0.54	* 0.75
hspA	-0.01	* 0.67	0.43	-0.05	* -0.83	-0.20	-0.43	0.56
hspC2	0.36	-0.39	-0.37	0.17	0.43	-0.37	0.64	-0.14
hspc4-1; hspc4-2	0.11	** 0.75	0.38	-0.12	-0.77	-0.43	-0.46	* 0.72
grp94	0.18	** -0.77	-0.55	0.42	** 0.93	0.06	* 0.67	-0.46
CLPB1	-0.19	0.17	0.50	0.33	-0.77	-0.26	0.27	0.25
dnaJ	0.23	* -0.69	-0.44	0.43	0.58	-0.55	** 0.86	-0.24
dnaK	-0.12	0.00	0.17	-0.69	0.03	0.09	-0.16	0.33
groL	-0.35	-0.27	0.47	0.40	-0.49	-0.54	0.45	-0.21
groL2	0.38	-0.03	-0.23	-0.52	0.26	0.20	-0.18	0.13
htpG	-0.53	-0.05	0.60	0.26	* -0.89	-0.09	0.21	0.05
iscU2	-0.08	-0.59	-0.15	-0.35	0.66	-0.20	0.39	-0.08
hydG	-0.45	0.00	0.52	0.29	-0.60	-0.31	0.36	-0.02

Spearman correlations		r value
*	p < 0.05	-1
**	p < 0.01	0
n =	6 to 11	1
