# Supplementary material for: Shifts in coastal sediment oxygenation cause pronounced changes in microbial community composition and associated metabolism
Source: Microbiome. 2017 Aug 9;5:96. doi: 10.1186/s40168-017-0311-5 (PMC5549381; doi:10.1186/s40168-017-0311-5)
Supplement: Supplementary file 8 [file 40168_2017_311_MOESM2_ESM.docx]

Sequencing facility ID	Sample name (16S rRNA gene)	Read pairs	Instrument setup (MiSeq)	Reads after merging	Reads after quality trimming	Amount of OTUs
P1130_2022	Water Oxic to Anoxic 1 day 12	49584	2×301 bp	46661	46015	228
P1130_2023	Water Oxic to Anoxic 2 day 12	110970	2×301 bp	57985	56713	456
P1130_2024	Water Oxic to Anoxic 3 day 12	66935	2×301 bp	56076	54829	698
P1130_2026	Water Oxic control 1 day 12	30208	2×301 bp	13458	13007	330
P1130_2027	Water Intermediate to Anoxic 2 day 12	72891	2×301 bp	62113	61079	1160
P1130_2028	Water Oxic control 2 day 12	80191	2×301 bp	58774	57464	471
P1130_2029	Water Intermediate to Oxic 3 day 12	64120	2×301 bp	54328	53137	941
P1130_2030	Water Intermediate to Oxic 2 day 12	68870	2×301 bp	59842	58613	1049
P1130_2036	Water Intermediate to Oxic 1 day 12	42756	2×301 bp	36705	35924	732
P1130_2037	Water Intermediate to Anoxic 1 day 12	78637	2×301 bp	70338	69225	1217
P1130_2038	Water Anoxic to Oxic 1 day 12	67278	2×301 bp	56272	55053	1492
P1130_2039	Water Anoxic control 1 day 12	88041	2×301 bp	84355	83223	1041
P1130_2040	Water Anoxic control 2 day 12	81417	2×301 bp	76068	74957	896
P1130_2042	Water Anoxic to Oxic 3 day 12	66933	2×301 bp	58664	57490	1482
P1130_2043	Water Anoxic to Oxic 2 day 12	67195	2×301 bp	58671	57367	1673
P1130_2044	Sediment Oxic field 1	74305	2×301 bp	58084	56569	2883
P1130_2045	Sediment Oxic field 2	51696	2×301 bp	35219	34138	1747
P1130_2046	Sediment Oxic field 3	80021	2×301 bp	60666	59150	2971
P1130_2047	Sediment Intermediate field 1	52187	2×301 bp	44262	43315	2228
P1130_2048	Sediment Intermediate field 2	56942	2×301 bp	45808	44638	2503
P1130_2049	Sediment Intermediate field 3	54604	2×301 bp	40041	39043	2088
P1130_2050	Sediment Anoxic field 1	67186	2×301 bp	44618	43318	2309
P1130_2051	Sediment Anoxic field 2	50394	2×301 bp	32078	31175	1639
P1130_2052	Sediment Anoxic field 3	61356	2×301 bp	49401	48107	2705
P1130_2053	Sediment Oxic to Anoxic 1	114566	2×301 bp	81844	79147	3111
P1130_2054	Sediment Oxic to Anoxic 2	52008	2×301 bp	45739	44483	2329
P1130_2055	Sediment Oxic to Anoxic 3	69638	2×301 bp	58628	56949	2534
P1130_2056	Sediment Oxic control 1	49278	2×301 bp	40788	39558	2141
P1130_2057	Sediment Oxic control 2	69953	2×301 bp	52557	51192	2786
P1130_2058	Sediment Intermediate to Oxic 1	36761	2×301 bp	30159	29331	1670
P1130_2059	Sediment Intermediate to Oxic 2	33694	2×301 bp	26712	26052	1392
P1130_2060	Sediment Intermediate to Anoxic 1	43510	2×301 bp	38853	37838	2311

P1130_2061	Sediment Intermediate to Anoxic 2	38666	2×301 bp	34482	33654	1787
P1130_2062	Sediment Intermediate to Oxic 3	56350	2×301 bp	52397	51175	2702
P1130_2063	Sediment Anoxic to Oxic 1	39511	2×301 bp	35326	34488	2057
P1130_2064	Sediment Anoxic to Oxic 2	44948	2×301 bp	41228	40253	2103
P1130_2065	Sediment Anoxic control 1	45338	2×301 bp	41453	40378	2384
P1130_2066	Sediment Intermediate to Anoxic 3	55014	2×301 bp	48829	47344	2893
P1130_2067	Sediment Anoxic control 2	33718	2×301 bp	28322	27548	1734
P1130_2068	Sediment Anoxic to Oxic 3	415814	2×301 bp	376267	366824	4649
P933_1035	Water Oxic field 1	38449	2×201 bp	2947	2329	180
P933_1036	Water Oxic field 2	8162	2×201 bp	1473	1118	143
P933_1037	Water Oxic field 3	138339	2×201 bp	46041	36507	386
P933_1038	Water Intermediate field 1	146232	2×201 bp	82144	65759	932
P933_1039	Water Intermediate field 2	126831	2×201 bp	73740	56959	1462
P933_1040	Water Intermediate field 3	231016	2×201 bp	140344	114655	1974
P933_1041	Water Anoxic field 1	191625	2×201 bp	106519	85795	1626
P933_1042	Water Anoxic field 2	202319	2×201 bp	111124	88465	1887
P933_1043	Water Anoxic field 3	48969	2×201 bp	9032	7135	69
P933_1044	Water Oxic to Anoxic 1 day 20	111072	2×201 bp	71859	59074	301
P933_1045	Water Oxic to Anoxic 2 day 20	95625	2×201 bp	22474	16717	235
P933_1046	Water Oxic to Anoxic 3 day 20	159886	2×201 bp	59431	47737	445
P933_1047	Water Oxic control 1 day 20	198488	2×201 bp	31619	25126	269
P933_1048	Water Intermediate to Anoxic 2 day 20	147322	2×201 bp	41135	32902	447
P933_1049	Water Oxic control 2 day 20	59309	2×201 bp	9336	6689	158
P933_1050	Water Intermediate to Oxic 3 day 20	103995	2×201 bp	57518	44399	536
P933_1051	Water Intermediate to Oxic 2 day 20	120436	2×201 bp	54685	42249	602
P933_1052	Water Intermediate to Anoxic 3 day 20	169361	2×201 bp	57502	45268	684

P933_1053	Water Intermediate to Oxic 1 day 20	174965	2×201 bp	2096	1590	218
P933_1054	Water Intermediate to Anoxic 1 day 20	146370	2×201 bp	6982	5496	334
P933_1055	Water Oxic to Anoxic 1 day 0	158175	2×201 bp	29812	22880	268
P933_1056	Water Oxic to Anoxic 2 day 0	55275	2×201 bp	7787	6117	153
P933_1057	Water Oxic to Anoxic 3 day 0	215418	2×201 bp	43281	34427	439
P933_1058	Water Oxic control 1 day 0	138814	2×201 bp	11702	9148	218
P933_1059	Water Intermediate to Anoxic 2 day 0	130372	2×201 bp	19139	15444	362
P933_1060	Water Oxic control 2 day 0	171115	2×201 bp	35016	28425	256
P933_1065	Water Anoxic to Oxic 1 day 20	38943	2×201 bp	1677	1325	134
P933_1066	Water Anoxic control 1 day 20	102436	2×201 bp	59062	50716	509
P933_1067	Water Anoxic control 2 day 20	103060	2×201 bp	61356	49679	497
P933_1068	Water Anoxic to Oxic 3 day 20	114548	2×201 bp	68971	52841	949
P933_1069	Water Anoxic to Oxic 2 day 20	174568	2×201 bp	35782	27138	303
P933_1074	Water Intermediate to Oxic 3 day 0	89233	2×201 bp	21936	17326	247
P933_1075	Water Intermediate to Oxic 2 day 0	30465	2×201 bp	19519	15673	976
P933_1076	Water Intermediate to Anoxic 3 day 0	109255	2×201 bp	16934	13174	214
P933_1077	Water Intermediate to Oxic 1 day 0	83045	2×201 bp	50813	41379	1638
P933_1078	Water Intermediate to Anoxic 1 day 0	81072	2×201 bp	53032	43120	1808
P933_1079	Water Anoxic to Oxic 1 day 0	65815	2×201 bp	41416	32102	1505
P933_1091	Water Anoxic control 1 day 0	200137	2×201 bp	41517	32013	677
P933_1092	Water Anoxic control 2 day 0	218795	2×201 bp	64903	51246	513
P933_1093	Water Anoxic to Oxic 3 day 0	212162	2×201 bp	51597	41010	472
P933_1094	Water Anoxic to Oxic 2 day 0	117985	2×201 bp	67477	53517	1564

Sequencing facility ID	Sample name (16S rRNA gene supplementary study)	Read pairs (MiSeq 2×301 bp)	Reads after merging	Read after quality trimming	Amount of OTUs
P2043_1087	Anoxic to oxic 1	193218	130090	99914	2611
P2043_1088	Anoxic to oxic 2	255217	170364	131005	2673
P2043_1089	Anoxic to oxic 3	303467	203119	156513	2644
P2043_1090	Anoxic to oxic 4	112266	72888	55596	2305
P2043_1091	Anoxic to oxic 5	200383	131186	99085	2861
P2043_1092	Anoxic Control 1	181706	117936	86811	2669
P2043_1093	Anoxic Control 2	187148	122350	90977	2731
P2043_1094	Anoxic Control 3	192579	126608	93217	2525
P2043_1095	Anoxic Control 4	214837	140467	104570	2769
P2043_1096	Anoxic Control 5	231661	151770	112419	2764

Sequencing facility ID	Sample name (metagenomics)	Million read pairs (MiSeq 2×301 bp)	Read pairs after quality trimming	Average read length after quality trimming	Contigs after assembly	Contig Min length	Contig Mean length	Contig Max length	N50	N50 length
P1611_201	Oxic to Anoxic 2	8,18	8085804	148	2324530	123	177	4294	943230	180
P1611_209	Oxic to Anoxic 3	9,08	8998502	141	2167366	123	182	25241	854344	183
P1611_217	Intermediate to Oxic 1	8,26	8192852	172	3481964	123	177	10757	1426845	180
P1611_225	Intermediate to Oxic 3	7,61	7516672	160	2676905	123	179	6469	1079404	182
P1611_233	Anoxic to Oxic 1	6,54	6477323	159	2641218	123	171	2676	1087325	172
P1611_241	Anoxic to Oxic 2	7,32	7084154	154	2848140	123	166	4348	1189661	167
	Combined assembly				3445144	123	193	14744	1271467	195

Sequencing facility ID	Sample name (metatranscriptomics)	Million read pairs (HiSeq 2×126 bp)	Read pairs after quality trimming	Average read length after quality trimming	Contigs after assembly	Contig Min length	Contig Mean length	Contig Max length	N50	N50 length
P2263_101	Oxic to Anoxic 2	40,90	21615896	126						
P2263_102	Oxic to Anoxic 3	43,36	21426238	126						
P2263_103	Intermeediate to Oxic 1	44,37	21077409	126						
P2263_104	Intermediate to Oxic 3	43,07	14758333	126						
P2263_105	Anoxic to Oxic 1	39,80	23332054	126						
P2263_106	Anoxic to Oxic 2	44,74	16430014	126						
P2263_107	Oxic Control 2	40,72	15637928	126						
P2263_108	Intermediate to Anoxic 2	43,23	18909459	126						
P2263_109	Intermediate to Anoxic 3	42,08	17614892	126						
P2263_110	Anoxic Control 1	44,92	18572975	126						
P2263_111	Anoxic Control 2	45,98	20817665	126						
	de-novo assembly				185092	201	389	17997	50135	387
